# Diverse *O*-methyltransferases catalyze the biosynthesis of floral benzenoids that repel aphids from the flowers of waterlily *Nymphaea prolifera*

**DOI:** 10.1093/hr/uhad237

**Published:** 2023-11-06

**Authors:** Guanhua Liu, Jianyu Fu, Lingyun Wang, Mingya Fang, Wanbo Zhang, Mei Yang, Xuemin Yang, Yingchun Xu, Lin Shi, Xiaoying Ma, Qian Wang, Hui Chen, Cuiwei Yu, Dongbei Yu, Feng Chen, Yifan Jiang

**Affiliations:** College of Horticulture, Nanjing Agricultural University, Nanjing 210095, China; Tea Research Institute, Chinese Academy of Agricultural Sciences, Hangzhou 310008, China; Tea Research Institute, Chinese Academy of Agricultural Sciences, Hangzhou 310008, China; Provincial Key Laboratory of Characteristic Aquatic Vegetable Breeding and Cultivation, Jinhua Academy of Agricultural Sciences (Zhejiang Institute of Agricultural Machinery), Zhejiang Province 321000, China; Provincial Key Laboratory of Characteristic Aquatic Vegetable Breeding and Cultivation, Jinhua Academy of Agricultural Sciences (Zhejiang Institute of Agricultural Machinery), Zhejiang Province 321000, China; College of Horticulture, Nanjing Agricultural University, Nanjing 210095, China; College of Horticulture, Nanjing Agricultural University, Nanjing 210095, China; Tea Research Institute, Chinese Academy of Agricultural Sciences, Hangzhou 310008, China; College of Horticulture, Nanjing Agricultural University, Nanjing 210095, China; Provincial Key Laboratory of Characteristic Aquatic Vegetable Breeding and Cultivation, Jinhua Academy of Agricultural Sciences (Zhejiang Institute of Agricultural Machinery), Zhejiang Province 321000, China; College of Horticulture, Nanjing Agricultural University, Nanjing 210095, China; Tea Research Institute, Chinese Academy of Agricultural Sciences, Hangzhou 310008, China; Graduate School of Chinese Academy of Agricultural Sciences, Beijing, 100081, PR China; Tea Research Institute, Chinese Academy of Agricultural Sciences, Hangzhou 310008, China; Graduate School of Chinese Academy of Agricultural Sciences, Beijing, 100081, PR China; Hangzhou Tianjing Aquatic Botanical Garden, Zhejiang Humanities Landscape Co., Ltd., Hangzhou 310000, China; Hangzhou Tianjing Aquatic Botanical Garden, Zhejiang Humanities Landscape Co., Ltd., Hangzhou 310000, China; Department of Plant Sciences, University of Tennessee, Knoxville, TN 37996, USA; College of Horticulture, Nanjing Agricultural University, Nanjing 210095, China

## Abstract

*Nymphaea* is a key genus of the ANA grade (Amborellales, Nymphaeales, and Austrobaileyales) of basal flowering plants, which serve as a key model to study the early evolution of floral traits. In this study, we comprehensively investigated the emission, biosynthesis, and biological function of the floral scent in a night-blossoming waterlily *Nymphaea prolifera*. The headspace volatile collection combined with GC–MS analysis showed that the floral scent of *N. prolifera* is predominately comprised by methylated benzenoids including anisole, veratrole, guaiacol, and methoxyanisole. Moreover, the emission of these floral benzenoids in *N. prolifera* exhibited temporal and spatial pattern with circadian rhythm and tissue specificity. By creating and mining transcriptomes of *N. prolifera* flowers, 12 oxygen methyltransferases (*NpOMTs*) were functionally identified. By *in vitro* enzymatic assay, NpOMT3, 6, and 7 could produce anisole and NpOMT5, 7, 9, produce guaiacol, whereas NpOMT3, 6, 9, 11 catalyzed the formation of veratrole. Methoxyanisole was identified as the universal product of all NpOMTs. Expression patterns of *NpOMTs* provided implication for their roles in the production of the respective benzenoids. Phylogenetic analysis of OMTs suggested a *Nymphaea*-specific expansion of the OMT family, indicating the evolution of lineage-specific functions. In bioassays, anisole, veratrole, and guaiacol in the floral benzenoids were revealed to play the critical role in repelling waterlily aphids. Overall, this study indicates that the basal flowering plant *N. prolifera* has evolved a diversity and complexity of OMT genes for the biosynthesis of methylated benzenoids that can repel insects from feeding the flowers. These findings provide new insights into the evolutional mechanism and ecological significance of the floral scent from early-diverged flowering plants.

## Introduction

The basal angiosperms comprise Amborellales (A), Nymphaeales (N), and Austrobaileyales (A), which is termed as ANA grade [1–3]. The complexity of floral scent emitted from the basal angiosperm flowers varies among different species [4, 5]. The flowers of *Amborella trichopoda*, the sole species of the *Amborellaceae* genus, are scentless [6], whereas the flowers of *Trimenia moorei* (a species of Austrobaileyales) emit a mixture of volatile compounds [7]. *Nymphaea* is an important ANA member and contains 45–50 species in five subgenera (*Anecphya*, *Brachyceras*, *Hydrocallis*, *Lotos*, and *Nymphaea*), which is globally distributed in four continents excepting Antarctica [1–3, 8–10]. As an aesthetic characteristic for ornamental application, the floral scent of *Nymphaea* plays pivotal role in attracting pollinators [10–12].

Based on the chemical structure and biosynthetic pathway, the floral volatile organic compounds (FVOCs) from 22 Nymphaeaceae species are generally classified as terpenoids, fatty acid derivatives, and benzenoids/phenylpropanoids [10, 11, 13–20]. The species-specific fatty acid methyl esters that are produced by the *SABATHs* were identified in *Nymphaea colorata* and *Victoria cruziana* as major floral scent compounds [10, 13]. For the terpenoid biosynthesis, sesquiterpenes released from the *N. colorata* flowers are synthesized by TPS-b members rather than the TPS-a members as in mesangiospermae, demonstrating the independent evolution of terpene synthase genes in *N. colorata* [10]. Volatile benzenoids/phenylpropanoids play crucial roles in attracting pollinators and natural enemies, activating disease resistance, and repelling pests through volatile signaling [21, 22]. Compared with the variation of terpenoids and fatty acid derivatives, benzenoids/phenylpropanoids have higher conservation among different *Nymphaea* species. Benzenoids/phenylpropanoids are detectable in the floral scent of all *Nymphaea* species and were identified as the major constituents in *N. amazonum*, *N. tetragona*, *N.lingulata*, *N. hybrida,* and *V. cruziana* [11, 13–15, 17–20]. The backbones of benzenoids/phenylpropanoids, including benzenoids (C6-C1), phenylpropanoid-related compounds (C6-C2), and phenylpropenes (C6-C3), are derived from phenylalanine by the shikimate pathway [23, 24]. The structural diversity of benzenoids/phenylpropanoids is attributed to the modification of the backbone, including hydroxylations, *O*-methylations, prenylations, and the formation of methylenedioxy bridges, which can activate or inactivate the biological functions of benzenoids/phenylpropanoids [23, 25]. Among these modifications, the methylation of benzenoids/phenylpropanoids changes their solubility in plant cells, which makes them hydrographic and easily volatilized [26].

Enzymatic methylation of metabolites is a ubiquitous way to regulate the biological activity of small and macromolecule metabolites of bacteria, fungi, plants, and animals [26, 27]. *O*-methylation of the plant hydroxy-methyl volatile compounds are catalyzed by *O*-methyltransferases (OMTs), which transfer the methyl group of S-adenosyl-L-methionine (SAM) to the hydroxyl group of an acceptor molecule with the formation of their corresponding methyl ether derivative and S-adenosyl-L-homocysteine as products [4, 27–29]. OMTs, which are responsible for the presence of volatile benzenoids/phenylpropanoids in the floral scent, have been extensively characterized in white campion (*Silene latifolia*) [30, 31], sweet basil (*Ocimum basilicum*) [32], anise (*Pimpinella anisum*) [33], loquat (*Eriobotrya japonica*) [34], rose (*Rosa hybrida*) [35], tomato (*Solanum lycopersicum*) [36, 37], and apple (*Malus domestica*) [38]. OMTs in plants are typically belonged to the type I family of methyltransferases because the methyl-group acceptors are secondary metabolites with small molecular mass [4]. A comprehensive analysis of protein sequences and the phylogenetic relationship of OMTs showed that the moderate number of conserved residues in OMTs of different clades could methylate similar substrates. Previous studies have shown that convergent evolution could be the driving force for OMTs to methylate a wide variety of substrates in mesagiospermae after initial duplication and evolutionary divergence [26]. However, the biosynthesis of benzenoids/phenylpropanoids by methylation and the evolution of OMTs in the floral scent of basal angiosperms have not been thoroughly explored.

Previous studies on the biological functions of floral volatiles in Nymphaeacea have mainly focused on its pollinator attractions [10–12, 15, 39, 40]. Flies and bees were recorded as the pollinators for daytime-blooming *Nymphaea* including subgenera *Brachyceras* and *Anecphya*, while night-blooming subgenera *Hydrocallis* and *Lotos* were observed to be specifically pollinated by beetles which synchronously consumed the floral tissues [40, 41]. The phenomenon of florivory as pollination in early angiosperm indicates the shift from mutualistic pollination interactions toward antagonistic relationships between flowers and insects during angiosperm evolution [42, 43]. For example, the visitation of *Trigona spinipe* driven by the consumption of floral tissues to the *Nymphaea pulchella* flowers strikingly improved the seed setting percentage [41]. Therefore, the floral scent plays key role in attracting pollinators for seed-bred waterlily, especially for the nocturnal blooming species [13, 39–41]. However, the biological function of floral volatiles emitted from vegetative waterlily species is still unexplored [40]. It will be tempting to investigate whether the floral scent in Nymphaeacea with vegetative propagation conserve the function in repelling florivores or attracting the natural enemy.

*Nymphaea prolifera*, an infertile species of Nymphaeales with a pristine habitat in South America, is a typical night-blossoming waterlily species that belongs to the subgenus *Hydrocallis* [44]. Interestingly, the plants of *N. prolifera* exhibited a unique reproductive style leading to vegetative propagation during development and cultivation. The early bloomed flowers originated from the rhizomes were termed as mother flowers. The subsequent bloomed flowers originated from mother flowers are defined as daughter flowers. Likewise, the lastly bloomed flowers originating from daughter flowers are defined as granddaughter flowers [44]. Finally, the granddaughter flower of *N. prolifera* develops the root system as a new plant [44]. In addition, as other night-blooming waterlilies [17], intense floral scent is perceptible from *N. prolifera* flowers during midnight anthesis by our olfactory system, while a subtle fragrance could be detected during the daytime. In this study, *N. prolifera* was selected as a model plant to investigate the biosynthesis and ecological function of floral scent in Nymphaeales. We present a comprehensive investigation of the chemical composition, emission pattern, and biosynthesis of benzenoids of *N. prolifera* flowers. The rhythmic emission of floral volatiles and the biochemical functions of OMTs that determine the formation of floral benzenoids in *N. prolifera* were investigated. Additionally, it was interesting to observe that *N. prolifera* plants are the hosts for waterlily aphids (*Rhopalosiphum nymphaeas*) in fields [45, 46] with more striking damage on the foliage or daughter/granddaughter flowers compared with the mother flowers. Therefore, the olfactory assay was performed to investigate the potential role of benzenoids in repelling waterlily aphids from mother flowers. These results will not only provide insights into the biology, biosynthesis and ecological function of the floral scent but also deepen our understanding of the origin and evolution of OMTs in Nymphaeacea.

## Results

### Identification of hydroxy-methyl benzenoids as the major floral volatile of *N. prolifera*

**Figure 1:**
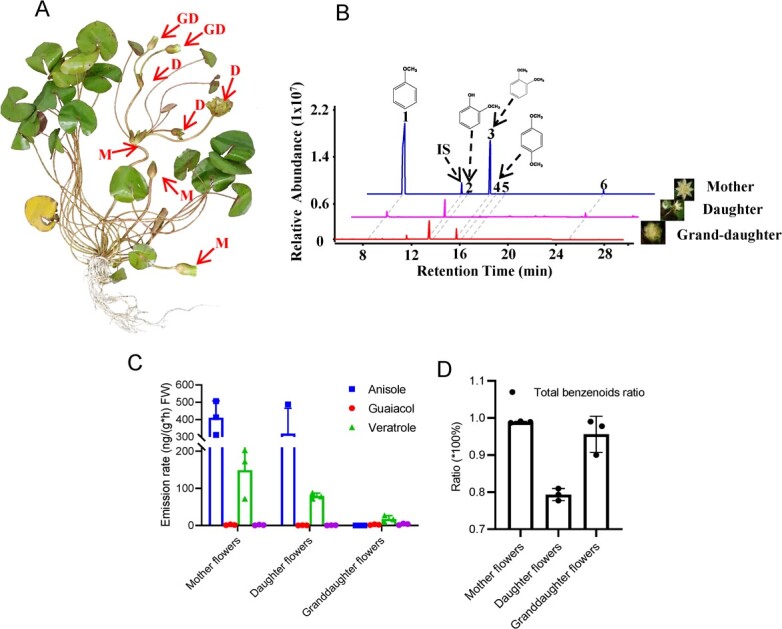
The floral volatile compound (FVOC) emission from different types of flowers in *Nymphaea prolifera*. **A** Different types of flowers based on the blooming time and growth characteristic in *N. prolifera*. M = mother flowers; D = daughter flowers; and GD = granddaughter flowers. **B** The chromatogram profile of FVOCs of the mother/daughter/granddaughter flowers of *N. prolifera* during the first blossom. Six detectable volatile compounds were marked in the first blossoming mother flowers. 1, anisole; 2, guaiacol; 3, veratrole; 4, methoxyanisole; 5, α-terpineol; and 6, pentadecane. The IS means the1-octanol as the internal standard. **C** The emission rate of four benzenoids of mother/daughter/granddaughter flowers during the first blossom. **D** The ratio of four major benzenoids in the total emission rate of FVOCs from mother−/daughter−/granddaughter flowers of *N. prolifera*.

Based on the growth characteristic of rhizomes, the first branching flowers from *N. prolifera* rhizomes were defined as mother flowers (M) (Fig. 1A). The flowers generated from the mother flowers were defined as daughter flowers (D) and the flowers produced from the daughter flowers were defined as granddaughter flowers (GD) (Fig. 1A). A total of six compounds were detected in the floral volatiles of the first-blossomed mother flowers of *N. profilera* (Fig. 1B; Table S1, see online supplementary material). Four benzenoid compounds (anisole, guaiacol, veratrole, and methoxyanisole) accounted for more than 95% of the total floral scent compounds of *N. prolifera* mother flowers (Fig. 1C and D, and2A). In the daughter flowers of *N. prolifera*, anisole, guaiacol, and veratrole were detected as the major constituents (Table S2, see online supplementary material). Moreover, guaiacol, veratrole, and methoxyanisole were also detected in the floral volatiles of granddaughter flowers during the 48 h observation period (Fig. 1B and C; Table S3, see online supplementary material). The emission rate of anisole was the highest in mother/daughter flowers (Fig. 1C). Intriguingly, the emission rate of veratrole, which was lower than the emission rate of anisole in mother/daughter flowers, was the highest in granddaughter flowers (Fig. 1C). These results indicated the variation of floral benzenoids among mother, daughter, and granddaughter flowers (Fig. 1B).

**Figure 2:**
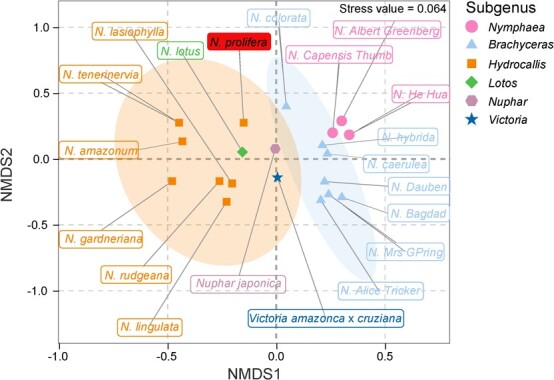
The NMDS (non-metric multidimensional scaling) analysis of FVOCs from *Nymphaea prolifera* mother flower with the other 20 species in Nymphaeaceae by R with vegan and ggplot2 packages. The detailed information of FVOCs in these Nymphaeaceae species was profiled in Table S5 (see online supplementary material).

The NMDS analysis of the FVOCs from 20 species in Nymphaeaceae illustrates significant variation among *Brachyceras*, *Nymphaea*, and *Hydrocallis* (Fig. 2). However, the profiling of FVOCs in *Nuphar* (*Nuphar japonica*), *Victoria* (*Victoria amazonica* × *cruziana*), and *Lotus* (*Nymphaea lotus*) were closed to *Hydrocallis* (Fig. 2). Moreover, the NMDS analysis further verified the species-specificity of anisole, guaiacol, veratrole, and methoxyanisole in Nymphaeaceae. It showed that the guaiacol, veratrole, and methoxyanisole were specifically presented in *N. prolifera*, whereas the anisole was specifically presented in *Hydrocallis* (Table S5, see online supplementary material).

### Rhythmic emission pattern of hydroxy-methyl benzenoids in floral scent of *N. prolifera*

**Figure 3:**
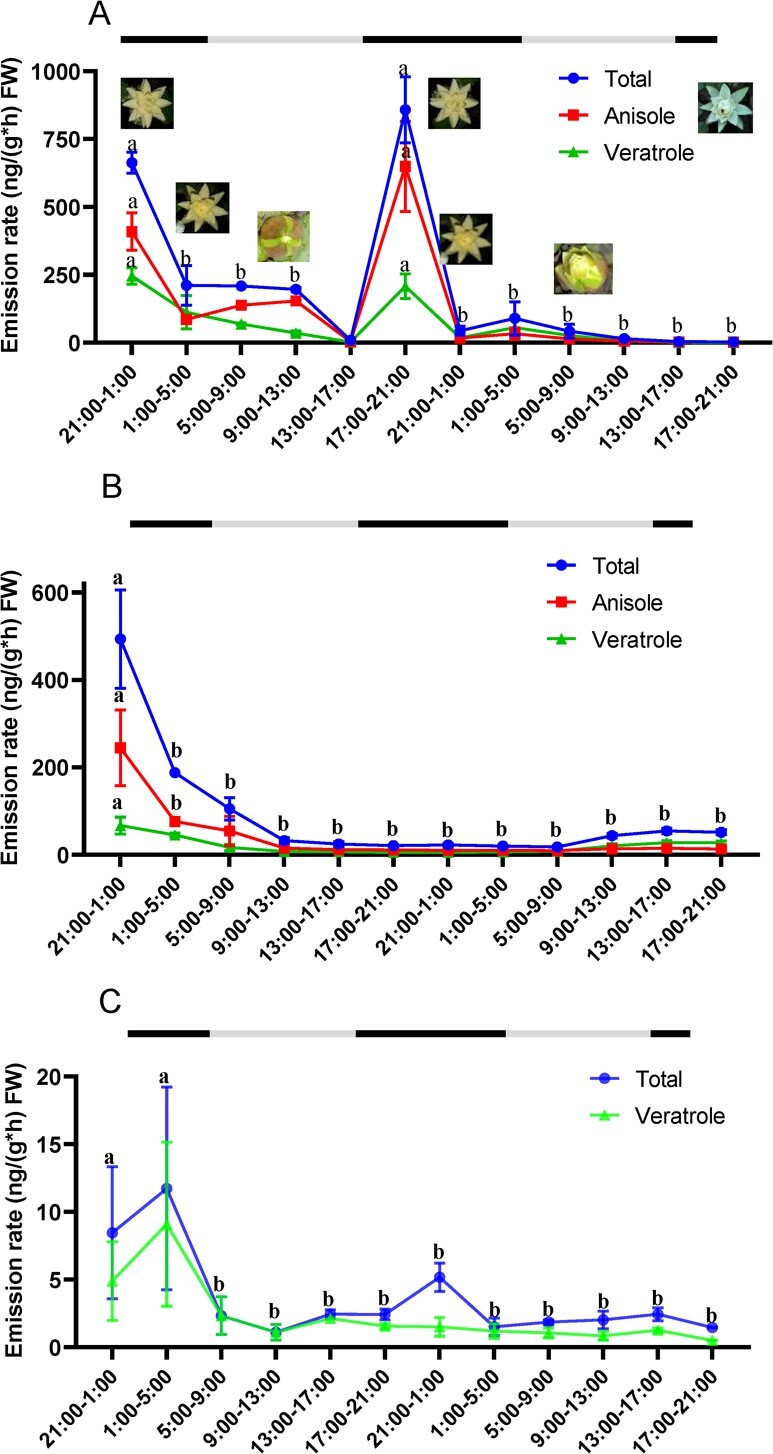
The rhythmic emission pattern of major volatile compounds of mother/daughter/granddaughter flowers during 48 h observation. **A** The emission pattern of total volatile compounds, anisole, and veratrole of mother flowers. **B** The emission pattern of total volatile compounds, anisole, and veratrole of daughter flowers. **C** The emission pattern of total volatile compounds and veratrole of granddaughter flowers. The black and gray bars indicate the nighttime and daytime, respectively. Different letters denote statistically significant differences among the mean values according to the analysis of variance (*P* < 0.05). The statistical test was conducted by Tukey-HSD by using SPSS (version 26).

The mother flowers of *N. prolifera* blossomed at 8 p.m. to 9 p.m. and closed at 5 a.m. to 6 a.m. during the dawn of the second day. During the second day, the mother flowers re-blossomed at 9 p.m. to 10 p.m. and closed at 5 a.m. to 6.30 a.m on the third day. On the third day, the mother flowers were tightly closed and submerged in water. During 48 h of observation, we collected FVOCs continuously at an interval of 4 h. The emission of total FVOCs and the two phenylpropenes, anisole and veratrole, from the mother flowers followed the same rhythmic pattern during the 48 h (Fig. 3A; Table S1, see online supplementary material). After re-blossoming, the emission rate of anisole and veratrole decreased drastically in the morning of the third day (Fig. 3A; Table S1, see online supplementary material).

The daughter/granddaughter flowers were constantly blossoming during the entire observation period. The emission pattern of total FVOCs, anisole, and veratrole from the daughter flowers was not rhythmic and decreased gradually from the first blossom to the third day (Fig. 3B; Table S2, see online supplementary material). The emission of veratrole in the granddaughter flowers was also not rhythmic and decreased gradually during the 48 h observation period (Fig. 3C; Table S3, see online supplementary material). Intriguingly, anisole, as the major volatile constituent of mother/daughter flowers, was not detected from granddaughter flowers during the observation period (Fig. 3B; Table S3, see online supplementary material).

### Spatially specific emission of hydroxy-methyl benzenoids in floral scent of *N. prolifera*

**Figure 4:**
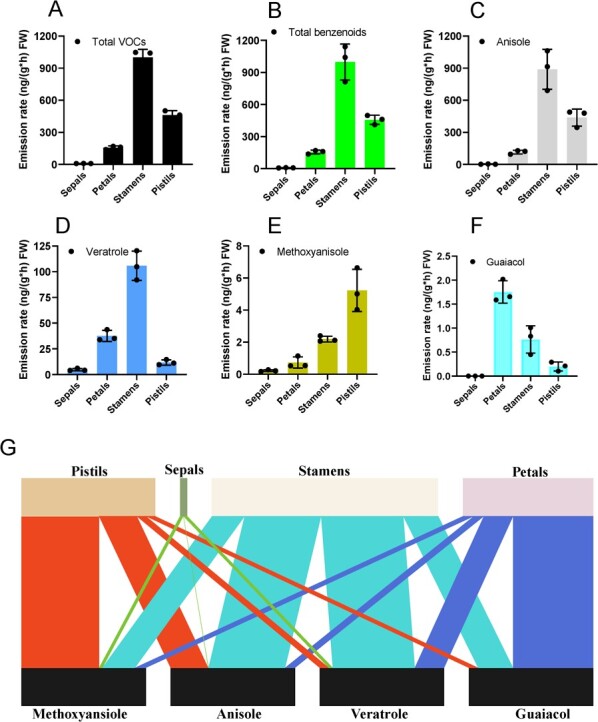
Emission rates of floral volatiles from different floral organs in *Nymphaea prolifera*. Intact fully opened mother flowers for the first time were separated into sepals, petals, stamens, and pistils and were subjected to dynamic headspace collection and GC–MS analysis, respectively. **A** The emission rate of total floral volatile compounds (FVOCs). **B** The total benzenoids in the four organs of *N. prolifera* mother flowers, respectively. **C**–**F** The emission rate of anisole, veratrole, methoxyanisole, and guaiacol in four floral organs (sepals, petals, stamens, and pistils). **G** The emission ratio of anisole, veratrole, methoxyanisole, and guaiacol from the four organs of mother flowers, respectively. The total VOCs and total benzenoids means that sum of all the compounds emission rate or all the benzenoids in each organ based on Table S4 (see online supplementary material), respectively. Bar widths correspond to the relative percentage of VOCs. The bipartite graph was constructed by R with bipartite package.

A total of 15 volatile compounds were detected in four floral organs of the *N. prolifera* mother flowers (sepals, petals, stamens, and pistils) with substantial variations (Table S4, see online supplementary material). The stamens and pistils were identified as the major emitter of the benzenoids (Fig. 4A). Anisole, veratrole, methoxyanisole, 6,9-heptadecadiene, and 8-heptadecene were found in all organs of the *N. prolifera* mother flowers (Table S4, see online supplementary material). The emission rate of three benzenoids (anisole, veratrole, and methoxyanisole) accounted for 81.81% of the total emission of the four organs of mother flowers (Fig. 4A and B; Table S2, see online supplementary material). Anisole, accounting for 58.89%–93.55% of the total emission in sepals and pistils, was detected as the major compound from the organs of the mother flower (Table S4, see online supplementary material). It is noted that 62% of the total anisole and 56% of the total veratrole emitted from the *N. prolifera* mother flowers were found to be derived from the stamens (Fig. 4B). All these results collectively indicated that benzenoids were the dominant FVOCs in the different organs of mother flowers (Fig. 4A and B).

### Identification of *NpOMT* genes from *N. prolifera* by transcriptomic analysis

A total of 12 *OMTs* were characterized from the transcriptome of the *N. prolifera* mother flowers (Accession No. PRJNA967977). The full length of all 12 NpOMTs (335-aa to 362-aa,) contained the conserved domains of typical plant OMTs (Fig. S1, see online supplementary material). Alignment of the deduced amino acid sequences of NpOMTs with biochemically characterized chavicol *O*-methyltransferase (CvOMT1) and (Iso)eugenol *O*-methyltransferase (EOMT1) of sweet basil showed that the metal ions combining the catalytically active site were highly conservative (Fig. S1, see online supplementary material) [32]. Some mutations of NpOMTs were found at SAM binding sites, and five motifs were contained in NpOMTs through Multiple Em for Motif Elicitation (MEME) analysis (Fig. S2, see online supplementary material).

### Characterization of the biochemical function of NpOMTs

**Figure 5:**
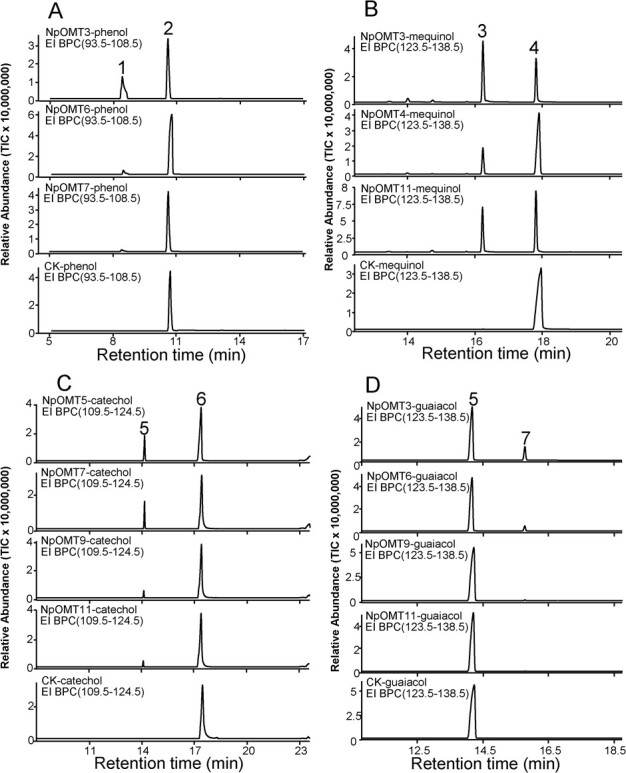
*In vitro* products catalyzed by *Nymphaea prolifera* oxygen methyltransferases (NpOMTs) with gas chromatography–mass spectrometry analysis. **A** The production of NpOMT3, NpOMT6, and NpOMT7 using phenol and S-adenosyl-L-methionine (SAM) as substrates. **B** The production of NpOMT3, NpOMT4, NpOMT11 using mequinol and SAM as substrates. **C** The production of NpOMT5, NpOMT7, NpOMT9, and NpOMT11 using catechol and SAM as substrates. **D** The production of NpOMT3, NpOMT8, NpOMT11, and NpOM13 using mequinol and SAM as substrates. The CK means the empty pET32a(+) as negative control. 1, anisole; 2, phenol; 3, methoxyaisole; 4, mequinol; 5, guaiacol; 6, catechol; 7, veratrole. All these seven compounds were confirmed by comparing the retention time and mass spectrum with their chemical standards.

Based on the chemical structure, we hypothesized that anisole is a methylated product of phenol. The enzymatic assays showed that NpOMT3, NpOMT6, and NpOMT7 function in the methylation of phenol to produce anisole (Fig. 5A and Table 1). Methoxyanisole was identified as the universal product of NpOMTs when incubated with SAM and mequinol (Fig. 5B and Table 1). Catechol was methylated by NpOMT5, NpOMT7, NpOMT9, and NpOMT11 to produce guaiacol, and the oxhydryl at the ortho-position of guaiacol was further methylated by NpOMT3, NpOMT6, NpOMT9, and NpOMT11 to produce veratrole (Fig. 5C and D and Table 1). Mequinol, detected in traces in mother flowers, was characterized as a product of NpOMT2 and NpOMT3 when incubated with hydroquinone and SAM (Fig. S3, see online supplementary material).

**Table 1:** Relative activity of recombinant NpOMTs with various substrates

Enzymes	Substrates				
	Phenol	Catechol	Guaiacol	Hydroquinone	Mequinol
NpOMT1					1.00 ± 0.066
NpOMT2				0.75 ± 0.133	1.00 ± 0.122
NpOMT3	0.55 ± 0.060		1.00 ± 0.024	0.50 ± 0.059	0.11 ± 0.010
NpOMT4					1.00 ± 0.171
NpOMT5		1.00 ± 0.016			0.04 ± 0.003
NpOMT6	0.30 ± 0.059		1.02 ± 0.013		0.11 ± 0.006
NpOMT7	0.014 ± 0.001	1.00 ± 0.106			0.034 ± 0.007
NpOMT8					1.00 ± 0.038
NpOMT9		1.01 ± 0.154	0.11 ± 0.019		0.12 ± 0.019
NpOMT10					1.00 ± 0.255
NpOMT11		1.00 ± 0.405	0.05 ± 0.003		0.105 ± 0.047
NpOMT12					1.00 ± 0.499

Note: All substrates were tested at the final concentration of 1 mmol/L. The relative activity of the products with the highest concentration were set as 100%. The relative activity of other products of the recombinant NpOMTs were obtained by the ratio of their concentrations with the product with highest concentration. For NpOMT1, NpOMT2, NpOMT4, NpOMT8, NpOMT10, and NpOMT12, the relative activity with mequinol was set arbitrarily as 100%. For NpOMT3 and NpOMT6, the relative activity with guaiacol was set arbitrarily as 100%. For NpOMT5, NpOMT7, NpOMT9, and NpOMT11, the relative activity with catechol was arbitrarily set as 100%.

### Expression patterns of the *NpOMT* genes in floral tissues of *N. prolifera*

**Figure 6:**
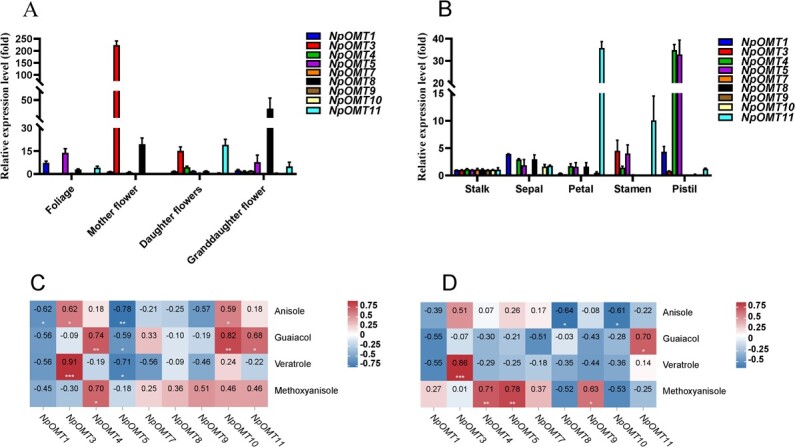
The relative expression analysis of *NpOMTs* in *Nymphaea prolifera* flowers, foliage, and stalk and correlation analysis of four major benzenoids with *NpOMTs* expression patterns. **A** The expression patterns of *NpOMTs* in foliage, mother/daughter/granddaughter flowers. **B** The expression patterns of *NpOMTs* in blooming floral organs of mother flowers. **C** The correlation analysis of *NpOMTs* expression patterns with four major benzenoids emission from mother−/daughter−/granddaughter flowers. **D** The correlation analysis of *NpOMTs* expression patterns with four major benzenoids emission from different organs in mother flowers. The gene transcript levels were measured using qRT-PCR with *NpActin* as the internal control, which was dug out from the transcriptome of *N. prolifera* mother flowers. The relative expression was evaluated using the 2^-ΔΔCT^ method. The correlation analysis was conducted by R studio with ggplot2 package. The numbers in the correlation map mean the relative coefficient.

As NpOMTs are responsible for the methylation of major floral volatiles, the expression patterns of 12 *NpOMTs* obtained from foliage, mother/daughter/granddaughter flowers, and five mother floral organs (stalk, sepals, petals, stamens, and pistils) were analysed by qRT-PCR using specific primers (Table S6, see online supplementary material). The varied expression patterns of 12 *NpOMTs* in foliage, mother flowers, daughter flowers, and granddaughter flowers were observed. The *NpOMT3* and *NpOMT8* were both highly expressed in mother or granddaughter flowers, while *NpOMT11* was highly expressed in foliage and granddaughter flowers (Fig. 6A). *NpOMT4* was highly expressed in daughter flowers (Fig. 6A). To further analyse the spatial expression pattern of *NpOMTs* in the mother flowers, the flowers were separated into five organs (stalk, sepals, petals, stamens, and pistils). The expression patterns of *NpOMTs* showed variations in the fully blossomed mother flowers of *N. prolifera* (Fig. 6B). The highest expression of *NpOMT1*, *NpOMT4*, and *NpOMT5* were observed in the pistils, whereas the highest expression of *NpOMT3* was observed in the stamens (Fig. 6B). *NpOMT11* was highly expressed in the petals and stamens of mother flowers (Fig. 6B). However, the expressions of *NpOMT2*, *NpOMT6*, and *NpOMT12* were not detectable in the foliage, stalk, mother flowers, and daughter/granddaughter flowers.

To further prove the role of *NpOMTs* in the benzenoids biosynthesis, the correlation between the *NpOMTs* expression and the emission of major benzenoids was established. The results indicated that the expression patterns of *NpOMT3* and *NpOMT4* were significantly correlated with the emission rate of anisole, veratrole (Fig. 6C and D), and methoxyanisole (Fig. 6C and D), respectively. Furthermore, the expression pattern of *NpOMT11* was positively correlative with the emission rate of guaiacol (Fig. 6C and D).

### Phylogenetic analysis of NpOMTs

**Figure 7:**
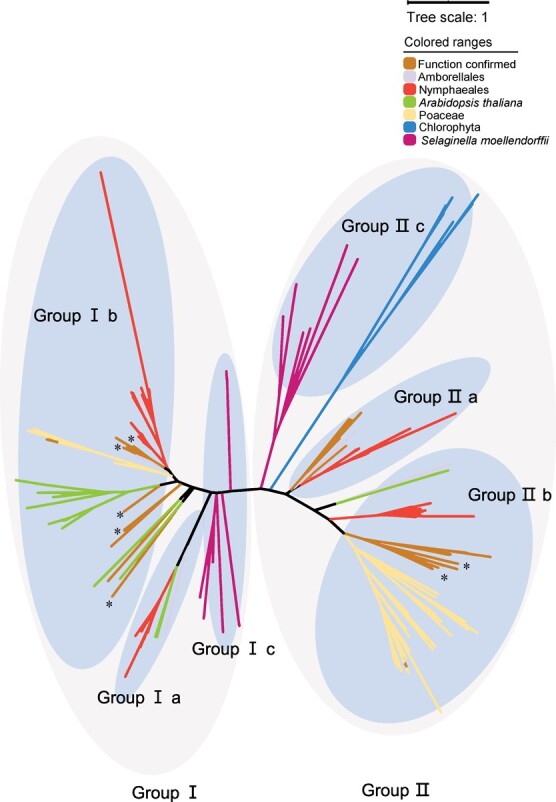
The phylogenetic analysis of NpOMTs with the putative OMTs identified from the genomes of *Amborella trichopoda*, *Nymphaea colorata*, *Arabidopsis thaliana*, *Sorghum bicolor*, *Euryale ferox*, *Selaginella moellendorffii*, and five *Chlorophyta* species, as well as functionally characterized OMTs from other plant species. Protein sequence alignments were produced by MEGA X, and the phylogenetic tree was constructed using the maximum likelihood method with 1000 bootstrap repetitions. The phylogeny was visualized by iTOL [49]. The putative OMTs of *N. colorata*, *E. ferox*, *S. bicolor*, *A. thaliana*, and *S. moellendorffii* were retrieved from NCBI by BlastP. The putative OMTs of the five Chlorophyta species were obtained from the UniProt TrEMBL protein database by referring to previous reports [50]. The functionally characterized OMTs represent that their catalyzed activities have been evaluated in previous reports and the detailed information is presented in Table S7 (see online supplementary material). “_*_” depicts OMTs involved in the biosynthesis of volatile hydroxy-methyl benzenoids/phenylpropanoids. Group I a, Group I b, and Group II c were distinguished based on substrates and sequence features. The detailed information on the functionally characterized OMTs is presented in Table S7 (see online supplementary material), and the detailed group information on the phylogenetic analysis is presented in Fig. S4 (see online supplementary material).

The putative OMTs identified from the genomes of *A. trichopoda*, *N. colorata*, *Arabidopsis thaliana*, *Sorghum bicolor*, *Euryale ferox*, *Selaginella moellendorffii*, and five *Chlorophyta* species, as well as functionally characterized OMTs from other plant species, were phylogenetically analysed with NpOMTs. Two divergent groups (I, II) of OMTs were obtained. The phylogenetic analysis showed that the function-identified small-molecule OMTs (smOMTs) of Nymphaeales (NpOMTs, NcOMTs, and EfOMTs) were grouped into six individual subgroups (Group I a, Group I b, Group II a, Group II b, Group I c, and Group II c; non-seed-plant-specific groups) with strong bootstrap support (Fig. 7; Fig. S4, see online supplementary material). NpOMT1–5 were clustered into Group II, whereas all other NpOMTs were clustered into Group I (Fig. 7; Fig. S4, see online supplementary material). NpOMT3,4,5, and NcOMT1,2,3,4,5,6,7,27 were grouped with AmtOMT2, AmtRetOMT2,3, and PtAEOMT (AAC49708.1, *Pinus taeda*) as Group II a, whereas NpOMT12, NcOMT28–33, and four EfOMTs were grouped with AmtFlaOMT and two caffeyl alcohol OMTs of *S. moellendorffii* as Group I a. Group II a and Group I a were defined as the basal specific OMTs of plants based on the phylogenetic analysis and substrate preferences (Fig. 7; Fig. S4, see online supplementary material) [47, 48]. OMTs from *Nymphaeales* were grouped independently with other angiosperm or gymnosperm SmOMTs, which were responsible for the biosynthesis of simple phenol, methylated caffeoyl-CoA, and flavonoids (Fig. 7). The OMTs in Nymphaeales exhibited a striking expansion compared with *A. trichopoda* OMTs (Fig. 7; Fig. S4 and Table S7, see online supplementary material). The *Nymphaeales* OMTs were clustered into four independent subclades, which were divergent with other plant OMTs, indicating the lineage-specific expansion of OMTs by repeated evolution (Fig. 7; Fig. S4, see online supplementary material).

### Behavioral responses of water lily aphids to floral odor cues

**Figure 8:**
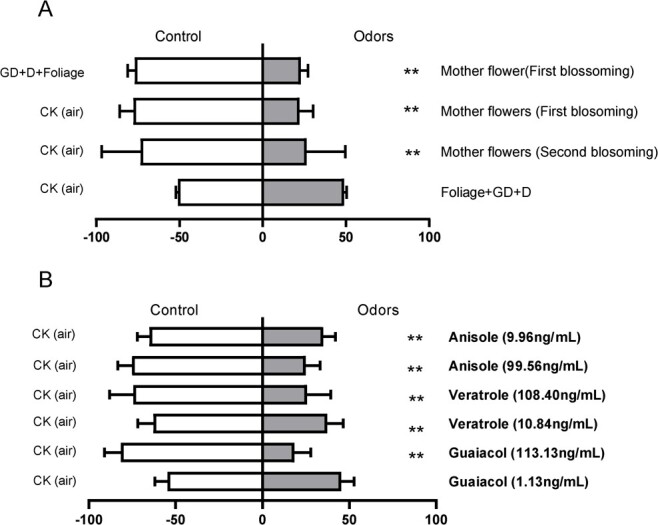
The preferences of aphids (*Rhopalosiphum nymphaeas*) to different odor resources. **A** The Y-tube assay for determining the preference of waterlily aphids to granddaughter/daughter flowers and foliage (GD + D + Foliage), mother flowers (first blossoming), and first closed mother flowers. **B** The preference assay of chemical standard (GC) anisole, veratrole, and guaiacol with respect to water lily aphids. The concentrations of anisole, veratrole, and guaiacol were selected based on the rhythmic emission ratio of mother flowers during the 24 h observation period. Control (air) was set with a solvent (paroline). Numbers refer to the percentage of the water lily aphids choosing an odor source. *N* is >54 per test. The total number of respondents involved in the selection was set as 100%. Significant differences were analysed using the chi-square test and are marked with asterisks (**P < 0.01; *P < 0.05).

In the field, *N. prolifera* was observed as a host for water lily aphids that feed only on foliage or daughter/granddaughter flowers but not mother flowers. The preferences of water lily aphids to different odors were evaluated by Y-tube assay (Fig. 8A). The results indicated that the aphids were repelled by the mother flowers of *N. prolifera* (χ^2^ = 28.02, *P* < 0.0001 for the first-blossomed mother flowers; χ^2^ = 68.74, *P* < 0.0001 for the closed mother flowers, Fig. 8A). Interestingly, there is no significant variation in the aphids preference between the granddaughter/daughter flowers and foliage and the air in the Y-tube assay setup (χ^2^ = 0.017, *P* = 0.089, Fig. 8A).

Based on the repelling effect of these mother flowers on the aphids, the repelling effect of three major floral volatile compounds (anisole, veratrole, and guaiacol) from *N. prolifera* was analysed. The results showed that the aphids were repelled by anisole and veratrole with the same emission rates as from mother flowers (χ^2^ = 84.07, *P* < 0.0001, χ^2^ = 103.58, *P* < 0.0001 for anisole and veratrole, respectively, Fig. 8B). However, the guaiacol had no effect on repelling or attracting the aphids at comparative low concentration (χ^2^ = 11.58, *P* = 0.07). With the concentration increasing to the natural emission rate from the flowers, the aphids were effectively repelled (χ^2^ = 8.38, *P* = 0.0038, Fig. 8B).

## Discussion

### Benzenoids emitted as major constituents from *N. prolifera* flowers with rhythmic patterns

Benzenoids are widely emitted from the floral scent and play vital roles in attracting pollinators and repelling florivores [38, 51]. Among diverse benzenoids, hydroxy-methyl benzenoids (anisole, guaiacol, veratrole, and methoxyanisole) were previously identified only in the floral scent of several specific angiosperm families, such as Arecaceae, Caryophyllaceae, Orchidaceae, and Magnoliaceae, mostly as a trace or minor constituent [16, 17, 52]. In the present study, anisole and veratrole were detected as major floral scent constituents in the mother flowers of *N. prolifera*, followed by guaiacol, methoxyanisole, and mequinol (Table S1, see online supplementary material). Besides *N. prolifera*, (methoxymethyl)-benzenes were also identified as dominant constituents in the floral scents of several *Nymphaea* species, such as *N. lingulata*, *N*. *lasiophylla*, *N. amazonum*, and *N*. *tenerinervia*, as well as a key compound in a more complex blend of *N. rudgeana* and *N. amazonum *subsp. amazonum [17]. The NMDS analysis of FVOCs from twenty Nymphaeaceae species indicated that the *N. prolifera* was classified with other *Hydrocallis* species due to the presence of species-specific benzenoids (Fig. 2; Table S5, see online supplementary material). Therefore, hydroxy-methyl benzenoids are produced as the specific category of volatile compounds in *Nymphaea*, reflecting the unique biosynthetic mechanism and evolutionary origin of floral benzenoids in Nymphaeales.

The blossoming and closing times of water lily flowers were typically rhythmic. In contrast, the emission patterns of floral scent in *V. cruziana* in *Nymphaeales* were non-rhythmic [13]. In the present study, the emission rates of anisole and veratrole from *N. prolifera* mother flowers decreased during the day and increased at night, which were synchronous with the rhythmic patterns of the flowering time (Fig. 3A). It is speculated that this rhythmic emission of benzenoids from *N. prolifera* could be regulated by the circadian clock to lure pollinators or deter florivores similar to other angiosperms [53, 54]. Furthermore, petals are major organs to emit benzenoids in a wide range of angiosperm species [13, 30, 34, 35, 55–61]. However, major benzenoids are emitted from the stamens and pistils of *N. prolifera* mother flowers (Fig. 4A and B). In addition, phenol could be in trace detected in the petals VOCs in the mother flowers (Table S4, see online supplementary material), indicating that massive accumulation of phenol in the petals.

### NpOMTs are responsible for the production of methylated benzenoids in *N. prolifera* flowers

OMTs, which transfer the methyl group of SAM to the hydroxy group of benzenoids, could be involved in the multiple steps of the methylation of benzenoid backbones [34]. For example, catechol was methylated by catechol-OMT to produce guaiacol, which was then methylated by guaiacol-OMT to produce veratrole in angiosperms [30, 31, 34, 36]. Our study presented the complete biosynthetic pathways of guaiacol and veratrole identified in *N. prolifera* (Fig. 5C and D andTable 1). Although anisole, mequinol, and methoxyanisole belonging to para-hydroxyl benzenoids have been identified in several species, their biosynthetic pathways are barely reported. To the best of our knowledge, ObCVOMT1 (Q93WU3.1) and ObCOMT1 (AAD38189.1) could methylate phenol with weak relative activity but not hydroquinone [32]. The present study showed that NpOMT3 efficiently methylated both substrates-phenol and hydroquinone (Table 1). As a metabolic intermediate, phenol is the simplest benzenoid containing only a hydroxyl group. To reduce its toxicity in plant cells, phenol is rapidly transformed into other compounds by modifications such as glycation [62]. *N. prolifera* produces anisole by methylating the only hydroxyl group of phenol, which reduces the toxic effect of phenol on cells and plays an important role in some other ecological functions, such as repelling the pest. Therefore, the methylation of phenol by NpOMT3,6,7 to produce anisole in *N. prolifera* flowers potentially indicated a novel metabolic pathway of benzenoids in basal angiosperm flowers.

**Figure 9:**
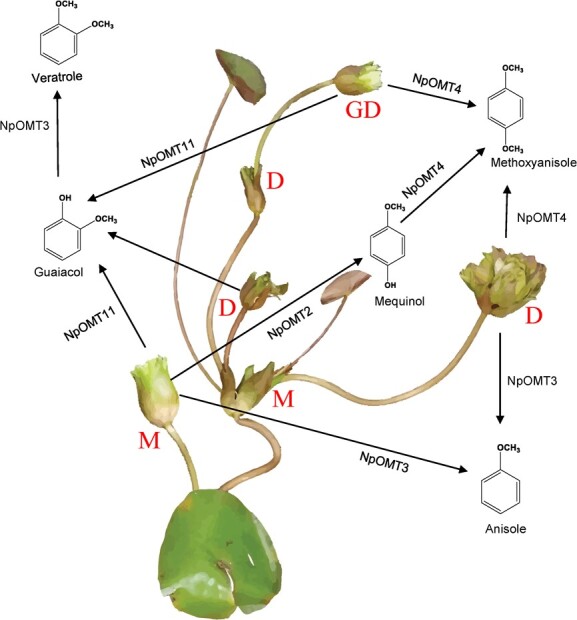
Proposed scheme for the biosynthesis of benzenoids in mother/daughter/granddaughter flowers of *Nymphaea prolifera*.

Regarding the substrate preference, the substrate specificity of NpOMTs was consistent with that of angiosperm OMTs, which can methylate a wide range of substrates as the multifunctional enzymes [26, 38]. Based on activity assay of NpOMTs and the correlation analysis between the expression patterns of *NpOMTs* (Fig. 6C and D) and emission rate of major benzenoids, we proposed a model of benzenoid methylation in *N. prolifera* flowers (Fig. 9). NpOMT3 could be mainly responsible for the methylation of mequinol to produce anisole, whereas NpOMT3 is responsible for the methylation of hydroquinone to produce mequinol. NpOMT11 is mainly responsible for the methylation of catechol to produce guaiacol. Veratrole is the main methylated product of NpOMT3 with guaiacol as a substrate. All 12 NpOMTs could produce methoxyanisole by methylating mequinol as substrate (Fig. 9 and Table 1). In addition, the correlation between the expression pattern of *NpOTM4* and methoxyanisole emission manifested that the NpOMT4 was responsible for methylating mequinol to generate methoxyansiole. However, NpOMT1,4,8,10,12 showed high specificity in terms of substrate preference, implying that they could produce O-methylated compounds detected in the floral volatiles of *N. prolifera* more readily than other non-volatile compounds.

### Lineage-specific expansion of *OMT* genes

OMTs in plants typically modify the chemical properties of hydroxyl phenylpropanoids, flavonoids, and alkaloids, thus playing roles in stress tolerance, lignin biosynthesis, and disease resistance [26]. In the present study, the biochemical functions of NpOMTs in Group II a and Group I a were different from those of the OMTs from other species with methylating caffeyl alcohol/5-hydroxyconiferyl alcohol in lignin biosynthesis, which was the ancient biochemical function of OMTs [26, 27, 47, 48, 63]. NpOMTs in Group II a and Group I a have evolved novel function in methylating the simple hydroxy group of phenylpropanoids such as phenol, catechol, and guaiacol. This analysis implied that the OMTs of Group II a and Group I a were the basal OMTs in angiosperms. The methylation of simple phenylpropanoids by NpOMT3,4,5 in Group II a and NpOMT12 in Group I a suggested that the OMTs of Nymphaeales have evolved novel function beyond lignin metabolism. The complex distribution of OMTs in Group II b and Group I b was consistent with the diverse biochemical functions of OMTs. Based on the phylogenetic analysis, OMTs catalyzing volatile benzenoids, such as SlCaOMT, RcEOMT, ObEOMT1, and RhOrOMT2, were intermixed with OMTs methylating isoflavones and caffeic acids, such as NtCaffOMT, ZmFOMT4, and MtIOMT7 [32, 35, 36, 61, 64–66]. In contrast, the majority of OMTs in Nymphaeales were classified into Group II a and Group I a. These results indicated that OMTs in Group II a and Group I a might retain the functions of their ancestor. The convergent or repeated evolution is the driving force for OMT evolution [26, 27, 67]. Additionally, genome duplication can drive the expansion of a gene family with a specific function [68]. The genome analysis of *N. colorata* and *E. ferox* indicated a Nymphaealean whole-genome duplication event, and polyploidization events were observed in Nymphaeaceae [10, 69, 70]. Compared with *A. trichopoda* OMTs, the intra-species expansion of OMTs in Nymphaeales was driven by recent, lineage-specific genome duplications [68]. The present study showed that all OMTs from Nymphaeales, Poaceae, and *S*. *moellendorffii*, as well as most OMTs from *A. thaliana* and *S. bicolor* were grouped in species-specific branches, manifesting the lineage-specific expansion of these OMTs with neofunctionalization (Fig. 7; Fig. S4, see online supplementary material) [71, 72]. Moreover, the conserved sequences of NpOMTs supported that these OMTs might have evolved from the same ancestor (Fig. S1, see online supplementary material). NpOMT3,4,5 and NpOMT7–11 that methylated phenol, catechol, and guaiacol were grouped as ancestral subclades, showing that the lineage-specific expansion drove the evolution of OMTs in *N. prolifera*.

### NpOMTs drive the competitive co-evolution

For core angiosperms, the floral scent performs multiple functions in luring pollinators, recruiting natural enemies, and resisting florivores [22, 24]. For some species in the ANA grade (such as *Trimenia moorei* and water lily), the floral scent specifically functions as the beacon for pollinators [7, 10–12, 15, 39, 73]. In some other ANA species (such as *A. trichopoda*, *Illicium dunnianum*, *I*. *tsangii*, *Schisandra henryi*, and *N. pulchella*), the flowers are also attractive to the insects for feeding or oviposition besides the pollinator attraction [6, 41, 74, 75]. The florivores simultaneously as pollinators for these basal angiosperms indicate that the mutualistic pollination interactions were evolved from antagonistic relationships between flowers and insects and these relationships shape the evolution of floral bouquets [42]. Benzenoids including isoeugenol, methyl benzoate, anisole, veratrole, guaiacol, and phenol have been reported to play the role as deterrent to florivores, sex pheromones, fumigants for controlling pests, and aggregation pheromones [76–81]. For example, the anisole and phenol were documented to orientate scarab beetles as sex pheromones [80, 82, 83]. In addition, the anisole, veratrole, and guaiacol could effectively attract the *Schistocerca gregaria* as aggregation pheromones [79]. Based on this evidence, we inferred that these emitted volatile benzenoids from *N. prolifera* mother flowers were generated from a shared metabolic pathway [84], could act as substances mimicking sex or aggregation pheromones to attract insects such as scarab beetles or desert locusts, which also serve as pollinators for the basal angiosperm flowers with simultaneous feeding.

The reproduction of seedless *N. prolifera* is mainly depended on vegetative propagation [42]. Thus, the floral scent from the *N. prolifera* could possess other significance in plant-organism interaction besides pollinator attraction. Compared with defense mechanisms of non-volatile compounds, employing volatiles as the cues to deter pest feeding can maximumly ensure the remote protection for plants [22, 85–87]. In the present study, the first-blossomed *N. prolifera* mother flowers effectively repelled the orientation of water lily aphids (Fig. 8C), which was not observed in daughter/granddaughter flowers and foliage (Fig. 8C). The massive emission of anisole, veratrole, guaiacol, and methoxyanisole were detected from the bouquet of daughter/granddaughter flowers. However, more than a half of the daughter/granddaughter flowers were submerged in water in the field, suggesting that the emission of the FVOCs of daughter/granddaughter flowers was restricted and isolated by water. In the preference assay for aphids, we simulated a situation with submerging daughter/granddaughter flowers below the water and floating mother flowers above the water. In the fields, it was observed that the damage of aphids to mother flowers was significantly less than that to daughter or granddaughter flowers, and leaves, which was consistent with our results of bioassay. These results clearly exhibit a conserved and primitive function of floral volatiles released from *Nymphaea* in florivores deterring. Furthermore, it has been demonstrated that tissue-specific emission of floral volatile compounds could maximumly resolve the dilemma about the carbon saving and the goal achieving in the pollinator attraction as well as the florivore repelling [88]. The tissue-specific emission pattern and repellent function against pest of floral volatiles were also observed in the mother-flowers of *N. prolifera* in our research (Figs 4 and8). According to the significantly distinct effects of floral volatile released from different tissues on the behavior of insects [88, 89], we postulated that the divergence of floral scent released by mother, daughter, and granddaughter flowers of *N. prolifera* also serve as the repellents to reduce the residence time and food intake of aphids as a strategy for flower protection [90]. In addition to tissue specificity, the floral scent emission of *N. prolifera* flowers also displayed the temporal fluctuation. The nocturnal blossoming waterlilies including *V. amazonica*, *Nymphaea rudgeana*, *N. amazonum* were frequently visited by the beetles for both consuming and pollinating [12, 15, 73]. By contrast, the flowers of some night-blooming species released massive benzenoids in the night to deter their florivores or attract the natural enemy of the florivores [22, 91]. Supported by this evidence, we postulate the rhythmic emission of floral benzenoids from *N. prolifera* mother flowers with the maximum emission in the night could also be related to repelling the nocturnal florivores (beetles) or attracting nocturnal predators of aphids (hoverflies). Our results underpinned the function of floral benzenoids from mother flowers in repelling the aphids, manifesting that the tissue-specific emission of benzenoids plays divergent roles in mediating the *N. prolifera* interaction with the same organism [88, 89].

It has been observed that seeds are rarely produced by *N. prolifera* flowers for breeding [44]. The infertility of *N. prolifera* flowers is probably attributed to the pollinator absence and pistils/stamen abortion. In contrast, the florivores which pollinated for the flowers of *N. prolifera* in their native-origin land, South America, or the introduction area are barely reported. Our bioassay results suggested that anisole, veratrole, and guaiacol emitted from *N. prolifera* mother flowers played crucial roles in repelling the aphids. Although aphids can adapt to volatile repellents to a certain extent, these repellents could successfully reduce the feeding frequency and residence time of aphids [90, 92]. On the other hand, the floral scent could also attract the natural enemies of the pest and play the function of an indirect defense [22]. For example, guaiacol, as one of the constituents detected from *N. prolifera* mother flowers, was recorded to attract *Coleomegilla maculata lengi* to predate the aphids as the ovipositional stimulants [93]. Thus, the role of major floral volatiles of *N. prolifera* in deterring aphids collectively manifested that the floral scent mainly functioned as a defense agent against antagonists or attraction of natural enemy for protecting *N. prolifera* flowers during vegetative propagation, rather than pollinator-attracting agents. In early angiosperms, these antagonists may be dominant due to the feeding and oviposition of insects on *A*. *trichopod* and *Austrobaileyales* flowers [6, 74, 75]. To summarize, anisole and veratrole, which were methylated by the independently evolved NpOMTs, played critical roles in the arming race for the competitive co-evolution relationship of florivores and early angiosperms.

## Conclusions

In this study, we present a comprehensive investigation of the chemical composition, emission regulation, and biosynthesis of the methylated benzenoids in *N. prolifera* flowers. The rhythmic patterns of floral volatile emission and the biochemical functions of OMTs in determining the modification of volatile benzenoids in basal angiosperm flowers were investigated. The emission rate of major benzenoids present in *N. prolifera* mother flowers was rhythmic, whereas the emission rate of benzenoids present in *N. prolifera* daughter/granddaughter flowers constantly decreased. Stamens and pistils were the major organs of mother flowers for benzenoid emission. NpOMT3, NpOMT6, NpOMT7, NpOMT2, and NpOMT1 were functionally characterized as the major OMTs for anisole, guaiacol, veratrole, and methoxyanisole biosynthesis in *N. prolifera*. The results suggested that convergent or repeated evolution could be the driving force for NpOMTs evolution based on the enzymatic assay with multiple substrates and phylogenetic analysis. The Y-tube assay revealed the repelling effect of anisole, veratrole and guaiacol on aphids, suggesting the competitive co-evolution relationship between *Nymphaea* and insects. The present study findings can provide a basis for understanding the floral scent biology of Nymphaeacea and deepen our understanding of the origin and evolution of OMTs in floral scent biosynthesis and ecological functions.

## Materials and methods

### Plant materials

Cloned *N. prolifera* plants were grown in Hangzhou Tianjing Aquatic Botanical Garden (E 120.12°, N 30.11°) under natural environmental conditions with regular management of fertilization, irrigation, disease prevention, and pesticide application. The full-blossomed flowers were harvested on the first day for floral volatiles collection.

### Collection and identification of floral volatiles

The floral volatiles emitted from *N. prolifera* flowers were collected using a dynamic headspace sampling system (Analytical Research System, Gainesville, FL, USA) as previously reported [94]. Volatiles were collected for 4 h by pumping clean air (0.8 L/min) from the glass chamber through a Super-Q (0.05 g) collection trap. The volatile compounds were eluted using 300 μL of dichloromethane containing 0.003% (*v*/*v*) 1-octanol as an internal standard. For the investigation in the emission rhythm of floral volatiles released from *N. prolifera* mother flower and daughter/granddaughter flower, each full-blossomed flower was sealed in a dynamic headspace sampling system for 48 h. The volatile compounds were collected at an interval of 4 h. The sepals, petals, stamens, and pistils were separated from the first blossoming mother flowers of *N. prolifera* and subjected to the FVOCs collection for 4 h. The collection and analysis of VOCs emitted from these floral organs were performed as described above. All the experiments were conducted with three replications.

The volatile compounds were analysed by the gas chromatography (GC)-mass spectroscopy (MS) instrument (Agilent Technologies 7890B) equipped with the chromatographic column of HP-5MS (30 m × 0.250 mm, 1909IS-433UI, Agilent Technologies. Inc, Carpinteria, California, USA). A splitless mode with an injection volume of 1 μL was applied with the injection temperature of 250°C. A gradient increasing of temperature with 5°C/min from 50°C (hold 3 min) to 250°C was employed. The electronic impact (EI) mode was set at 70 eV. All the data of mass spectral from 40 to 400 m/z were obtained [94]. The emission rate was calculated as previously described [13]. All compounds were identified using the National Institute of Standards and Technology mass spectral database by comparing the retention times and mass spectra with available chemical standard compounds (https://www.nist.gov/srd).

### Mining the *NpOMT* genes from the flowers of *N. prolifera* by transcriptomics

The putative NpOMTs were obtained from the transcriptome of *N. prolifera*, with ObCOMT (AF435007.1) as a template for protein query. The OMTs were identified from *N. colorata*, *E. ferox, A. thaliana, S. bicolor*, five Chlorophyta species, and *S. moellendorffii* genome by using the same methodology. The putative NpOMTs, NcOMTs, and EfOMTs were further manually annotated by searching the National Center for Biotechnology Information (NCBI) nonredundant protein sequence database by BlastP (https://blast.ncbi.nlm.nih.gov/Blast.cgi) with default parameters. The typical plant-originated OMTs for sequence alignments were retrieved using BlastP from NCBI (https://blast.ncbi.nlm.nih.gov/Blast.cgi), and detailed information is listed in Table S7 (see online supplementary material). For phylogenetic and multiple sequence analyses, the OMTs from different species were obtained through BlastP using default parameters, and the organism was chosen from the NCBI database (https://blast.ncbi.nlm.nih.gov/Blast.cgi). Detailed information on all the polygenetic analysis sequences is presented in Table S7 (see online supplementary material). Sequence alignment of OMTs was performed using the ClustalW program embedded in MEGA X. A phylogenetic tree was also constructed by MEGA X based on maximum likelihood (ML) using Jones-Taylor-Thornton (JTT) model through bootstrapping with 1000 replicates [95].

### Biochemical activity assay of NpOMTs

Full-length open reading frames of 12 *NpOMT* genes were synthesized (Genescript, Nanjing) and subcloned into the vector pET32a(+) (http://www.emdmillipore.com) based on the original transcriptome sequences. The recombinant NpOMT-pET32a(+) plasmids were transformed into *Escherichia coli* (BL21 Condon plus) by heat shock at 42°C for 1 min. After the cells were rejuvenated at 37°C, 225 rpm for an hour in LB liquid medium, they were cultured to an OD600 of 0.6 in 50 mL LB liquid medium with 50 μg/mL ampicillin at the same condition. Protein expression was induced by 1 mM IPTG (isopropyl-β-D-thiogalactopyranoside, 50 mg/mL, Tiangen, Ltd., Beijing, China) at 18°C and 180 rpm for 18 h. The cells were harvested by centrifugation and sonicated for 6 × 30 sec in chilled extraction buffer: 100 mM Tris–HCl (pH = 7.2), 5 mM MgCl_2_, 10 mM β-mercaptoethanol, and 10% glycerol (*v*/*v*). The crude enzymes were assayed for methyltransferase activities. An empty pET32a(+) plasmid without any insert was used as a negative control.

To identify the catalytic products of NpOMTs, crude protein was combined with 1 μM substrates (guaiacol, phenol, mequinol, catechol, or hydroquinone) in reaction buffer (1 μM of unlabeled SAM, 100 mM Tris–HCl [pH = 7.2], 5 mM MgCl_2_, 10 mM β-mercaptoethanol, and 10% glycerol [*v*/*v*]) in 200 μL reactions at 37°C for 1 h. Every NpOMT was tested all the five substrates (guaiacol, phenol, mequinol, catechol, or hydroquinone) as the methyl receptor and the SAM as the methyl donor, each substrates test for three repetitions. The reaction products were collected using ethyl acetate with 0.003% (*v*/*v*) n-octanol as an internal standard and analysed by GC–MS^31^. All the products were detected by subjecting the organic layer to GC–MS (Agilent Technologies 7890B GC–MS system) with the same protocol as the FOVCs analysis. The volatile concentration was calculated with the following formula:


\begin{equation*}PC={\frac{Sp}{Si}}\,{\ast}\,I \end{equation*}

*PC* and *I* represent the concentration of product and internal standard, respectively. *Sp* and *Si* indicate the peak area of the individual product and internal standard, respectively. The relative activity of the products with the highest concentration were set as 100%. The relative activity of other products of the recombinant NpOMTs were obtained by the ratio of their concentrations with the product with the highest concentration.

### Tissue-specific expression of *NpOMTs*

Total RNAs from intact mother/daughter/granddaughter flowers and foliage, flower stalks, sepals, petals, stamens, and pistils of the first-blossomed mother flowers were extracted using the RNAprep Pure Plant Plus Kit (Polysaccharides & Polyphenolics-rich, TIANGEN Ltd, Beijing) following the manufacturer’s protocols, and then reverse-transcribed into cDNA by using the PrimeScript™ RT reagent Kit with gDNA Eraser (Perfect Real Time) (Takara, Co. Ltd, Dalian, China) following the manufacturer’s protocol. Gene transcript level was investigated by quantitative reverse transcriptase (qRT)-polymerase chain reaction (PCR) with aliquots of 200 ng cDNA and 10 ng primers, and at least three biological repeats were run each time. The sequences of the internal standard (*NpActin*) primers were designed based on the transcriptome of the *N. prolifera* mother flowers and confirmed by RT-PCR. Detailed information on the primers is given in Table S6 (see online supplementary material). qRT-PCR was performed following the manual of SYBR Premix Ex Taq II Kit (Takara, China) using an ABI 7500 Real-Time PCR System (Applied Biosystems, CA, USA). Relative gene expression was calculated using the 2^−ΔΔCT^ method based on the expression of all *NpOMTs* in the stalk, which was set as previously described [96]. The statistical significance of the relative gene expression was analyzed by performing Wilcoxon’s two-group test by SPSS software (version 26).

### Behavior responses of *R*. *nymphaeas* to odor sources

Wild *R. nymphaeas* were collected from the water lily pond in Jinhua City, Zhejiang Province. All the aphids were raised in an insectary (temperature of 25 ± 2°C; relative humidity of 60%–70%) with the foliage of *N. prolifera* as the food. Behavioral response of *R. nymphaeas* to VOCs from mother/daughter/granddaughter flowers, foliage, and chemical standards (including anisole, veratrole, and guaiacol) were analysed using a Y-shape olfactometer composed with a 12 cm glass base tube (diameter = 1.5 cm) combining with two 12 cm glass arm tube (diameter = 1.5 cm) at the angle of 75°. Each glass arm tube was connected to a container with odor source, which is equipped with a humidifier bottle, a filtering tube filled with activated charcoal, and a rotameter. Both arms were connected to the exhaust of a pump. The airflow was controlled by the rotameter at 400 mL/min. The water lily aphids were allowed to move through the tube base toward the odor sources. The Y-tube olfactometer was placed in a uniformly illuminated room with the temperature constantly maintained at 24–26°C. Each aphid was singly introduced to enter the base tube for each replication. When an aphid crossed a line at 5 cm after the division of the base tube and stayed for at least 1 min, the behavior as positive choice toward the odor was recorded. After eight repetitions, the olfactometer tube was washed with 80% alcohol followed by the heating at 100°C for 20 min. The position of the containers with odor source were reversed after each replication to eliminate directional bias. The connections of the odor source glass tube to the rotameter were washed with 80% alcohol followed by heating at 100°C for 20 min after every replication to avoid contamination. The chemical standard (anisole, veratrole, and guaiacol) were dissolved in paroline.

### Statistical analysis

Differences in the emission rates of total FVOCs, individual benzenoids among different floral organs, and the emission rhythm of FVOCs were analysed by one-way analysis of variance (ANOVA) using SPSS software (version 26). All statistical effects were considered significant at *P* < 0.05. All *P* values for multiple comparisons were corrected by Bonferroni correction. The statistical significance of gene relative expression was analysed using SPSS software (Version 26). Bar and pie graphs were constructed by GrapPad Prism 9 (GraphPad Software, USA). The significant differences in aphid selection by the Y-tube assay were analysed by the chi-square detection test by SPSS software (version 26). The correlation analysis with Pearson model was conducted online with Omicshare (https://www.omicshare.com/tools/). The NMDS (non-metric multidimensional scaling) plot was produced by R studio with vegan and ggplot2 packages.

## Supplementary Material

Web_Material_uhad237

## Data Availability

All data generated or analysed in this study are included in this published article and its supplementary information files.
