# Supplementary material for: Diverse O-methyltransferases catalyze the biosynthesis of floral benzenoids that repel aphids from the flowers of waterlily Nymphaea prolifera
Source: Hortic Res. 2023 Nov 6;10(12):uhad237. doi: 10.1093/hr/uhad237 (PMC10753166; doi:10.1093/hr/uhad237)
Supplement: Web_Material_uhad237 [file web_material_uhad237.zip › Supplementary Figures and Tables - HR - 20230914.docx]

**
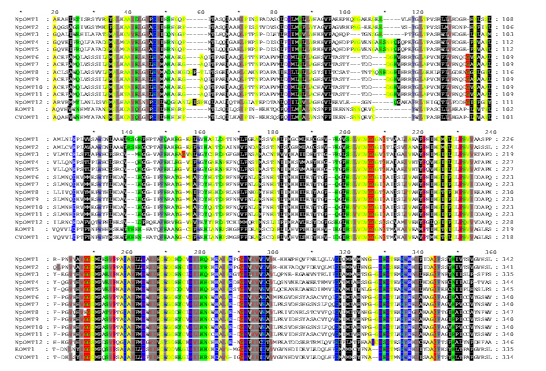
**

**Fig. S1 The alignment of NpOMTs with chavicol *O*-methyltransferase (CVOMT1, Q93WU3.1) and (Iso)eugenol** ***O*-methyltransferase (EOMT1, Q93WU2.1) of sweet basil (*O. basilicum*).** The purple, cyan and red underline indicated metal ion binding site, catalytically active site and SAM binding site, respectively.

**
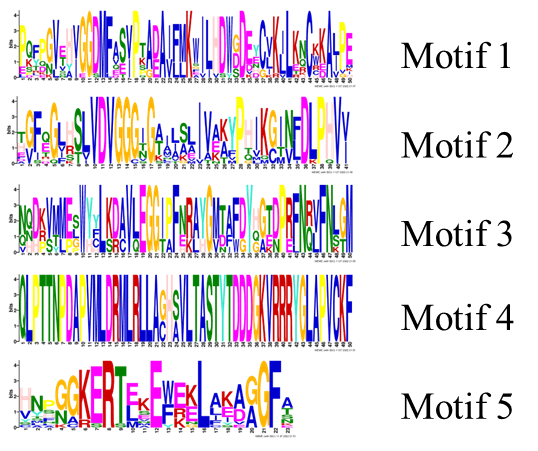
**

**Fig. S2 The motif analysis of NpOMTs by MEME (**[**https://meme-suite.org/meme/tools/meme**](https://meme-suite.org/meme/tools/meme)**) with default parameters.**

**NpOMT3-Hydroquinone**

**0**

**2**

**4**

**6**

**10**

**15**

**20**

**25**

**0**

**2**

**4**

**6**

**2**

**4**

**6**

**EI BPC (109.5-124.5)**

**NpOMT2-Hydroquinone**

**EI BPC (109.5-124.5)**

**CK-Hydroquinone**

**EI BPC (109.5-124.5)**

**0**

**Relative Abundance (TIC x 10,000,00)**

**1**

**2**

**Fig. S3 Products analysis by GC-MS of the reaction catalyzed by NpOMT2 and NpOMT3 incubation with hydroquinone.** The reaction products of NpOMT2 and NpOMT3 by using hydroquinone and SAM as substrates. 1, mequinol; 2, hydroquinone.





**Group Ⅱ**

**Group Ⅰ**

**Group Ⅱ c**

**Group Ⅱ a**

**Group Ⅱ b**

**Group Ⅰ c**

**Group Ⅰ a**

**Group Ⅰ b**

**Fig. S4. A rooted phylogenetic tree of plant *O*-methyltransferase**. Protein sequences used in this analysis are the same as those used for making Figure 6. The abbreviated gene names are the biofunction confirmed OMTs based on previous reports, and detail information about these genes were filed in Table S6. The blue label back ground means the substrates of these OMTs are Isoflavone or caffeic acid whose products are non-volatile benzenoids. The yellow label back ground means that the substrates of these OMTs are guaiacol, catechol or eugenol whose products are volatile benzenoids.

**Table S1.** The FVOCs from the mother flowers of *N. profilera*.

| **Compounds (μg/g FW^-1^*4 h)** | **21:00-1:00** | **1:00-5:00** | **5:00-9:00** | **9:00-13:00** | **13:00-17:00** | **17:00-21:00** | **21:00-1:00** | **1:00-5:00** | **5:00-9:00** | **9:00-13:00** | **13:00-17:00** | **17:00-21:00** |
| --- | --- | --- | --- | --- | --- | --- | --- | --- | --- | --- | --- | --- |
| Anisole | 408.87±137.49a | 85.11±8.88b | 137.70±3.91b | 154.05±27.22b | 3.42±0.004b | 649.47±333.31a | 17.16±19.64b | 32.50±42.72b | 13.79±16.75b | 5.17±3.37b | 2.77±0.57b | 1.71±0.83b |
| Guaiacol | 0.82±1.04 | 0.20±0.24 | ND | ND | ND | ND | 1.68±2.08 | ND | ND | ND | ND | 0.21±0.08 |
| Bicyclo[2.2.2]octan-1-ol, 2-methyl- | ND | ND | 0.17±0.19 | ND | ND | ND | ND | ND | ND | ND | ND | ND |
| Linalool | ND | ND | ND | ND | 0.26±0.08 | ND | ND | ND | ND | ND | ND | ND |
| Veratrole | 245.25±60.11a | 111.82±123.54 | 68.57±8.49 | 35.63±14.68 | 2.05±0.04 | 208.10±90.70 | 17.84±20.50 | 55.10±77.31 | 26.16±34.35 | 6.01±3.71 | 0.97±0.06 | 0.34±0.12 |
| Methoxyanisole | 0.67±0.144 | ND | 0.17±0.01 | 0.95±0.38 | ND | 0.39±0.06 | ND | 0.12±0.06 | ND | 0.12±0.15 | 0.08±0.10 | 0.07±0.004 |
| Mequinol | ND | ND | ND | ND | ND | ND | ND | 0.02±0.02 | ND | ND | ND | ND |
| Tridecane | ND | ND | ND | ND | ND | ND | 0.05±0.01 | ND | ND | ND | ND | ND |
| 1,2-Benzisothiazole | ND | 0.22±0.30 | ND | ND | ND | ND | ND | ND | ND | ND | ND | 0.79±0.11 |
| α-Terpineol | 0.62±0.18 | ND | ND | 0.22±0.13 | ND | ND | ND | ND | ND | ND | ND | ND |
| Pentadecane | 6.58±1.20 | 4.18±3.16 | 1.95±1.57 | 5.67±0.61 | 1.73±0.62 | ND | 3.82±5.28 | 1.43±1.79 | 1.66±1.61 | 1.91±1.40 | 0.25±0.04 | 0.14±0.03 |
| α-Farnesene | ND | 1.87±2.42 | ND | ND | 0.97±0.49 | ND | ND | ND | ND | ND | ND | ND |
| Hexadecane | ND | ND | ND | ND | ND | ND | 0.38±0.07 | ND | ND | ND | ND | ND |
| Cedrol | ND | ND | ND | ND | ND | ND | 0.11±0.01 | ND | ND | ND | ND | ND |
| 6,9-Heptadecadiene | ND | 5.95±6.93 | 0.12±0.13 | ND | ND | ND | ND | ND | 0.61±0.82 | 0.89±1.10 | ND | ND |
| 8-Heptadecene | ND | 1.78±1.01 | 0.15±0.18 | ND | ND | ND | 2.31±2.92 | 0.04±0.03 | 0.15±0.17 | 0.27±0.18 | ND | ND |
| Heptadecane | ND | ND | ND | ND | ND | ND | 0.51±0.39 | ND | ND | ND | ND | ND |

Note: ND, means non detected. The letters, a,b, means the ANOVA analysis.

**Table S2.** The FVOCs from the daughter flowers of *N. profilera*.

| Compounds ((μg/g FW^-1^*4 h) | 21:00-1:00 | 1:00-5:00 | 5:00-9:00 | 9:00-13:00 | 13:00-17:00 | 17:00-21:00 | 21:00-1:00 | 1:00-5:00 | 5:00-9:00 | 9:00-13:00 | 13:00-17:00 | 17:00-21:00 |
| --- | --- | --- | --- | --- | --- | --- | --- | --- | --- | --- | --- | --- |
| Anisole | 366.38±172.56 | 113.31±15.58 | 79.96±67.09 | 21.10±6.64 | 14.85±4.74 | 13.82±0.18 | 11.73±0.61 | 11.41±1.77 | 10.06±0.03 | 15.38±0.92 | 16.73±3.07 | 13.82±2.91 |
| Guaiacol | 2.09±1.93 | 0.37±0.18 | 0.26±0.02 | 0.11±0.09 | 0.19±0.02 | 0.17±0.03 | 0.19±0.04 | 0.44±0.002 | 0.37±0.03 | 1.28±0.36 | 1.80±0.63 | 1.96±0.88 |
| Veratrole | 99.55±38.88 | 66.65±8.56 | 23.47±15.18 | 8.88±6.19 | 7.63±5.98 | 5.51±3.24 | 4.69±3.46 | 5.61±3.38 | 7.18±5.57 | 25.73±10.12 | 35.61±9.85 | 35.14±9.08 |
| Methoxyanisole | 3.11±1.05 | 1.13±0.62 | 0.28±0.05 | 0.08±0.02 | 0.04±0.01 | 0.04±0.01 | 1.63±2.18 | 2.54±3.50 | ND | 0.33±0.05 | 0.48±0.09 | 0.51±0.14 |
| Pentadecane | 22.36±10.45 | 6.12±6.12 | 1.02±1.19 | 2.19±0.13 | 2.08±0.06 | 1.34±0.85 | 4.14±4.41 | ND | ND | ND | ND | ND |
| Anisole ratio | 0.7395±0.0127 | 0.6028±0.0266 | 0.6875±0.3071 | 0.6636±0.0590 | 0.6157±0.0768 | 0.6659±0.0777 | 0.5418±0.1261 | 0.5689±0.0418 | 0.6016±0.1919 | 0.3617±0.0658 | 0.3077±0.0125 | 0.2688±0.0007 |
| Veratrole ratio | 0.2051±0.0147 | 0.3547±0.0124 | 0.2925±0.2858 | 0.2568±0.0890 | 0.2819±0.1185 | 0.2573±0.1282 | 0.1955±0.0991 | 0.2743±0.1463 | 0.3767±0.1971 | 0.5806±0.0932 | 0.6481±0.0355 | 0.6800±0.0314 |

Note: ND, means non detected.

**Table S3.** The FVOCs from the grand-daughter flowers of *N. profilera*.

| Compounds ((μg/g FW^-1^*4 h) | 21:00-1:00 | 1:00-5:00 | 5:00-9:00 | 9:00-13:00 | 13:00-17:00 | 17:00-21:00 | 21:00-1:00 | 1:00-5:00 | 5:00-9:00 | 9:00-13:00 | 13:00-17:00 | 17:00-21:00 |
| --- | --- | --- | --- | --- | --- | --- | --- | --- | --- | --- | --- | --- |
| Sabinene | ND | ND | ND | ND | ND | ND | 0.62±0.20 | ND | 0.33±0.11 | ND | ND | ND |
| Guaiacol | 0.81±0.22 | ND | ND | ND | ND | ND | 2.09±0.71 | ND | 0.46±0.10 | 0.95±0.23 | 1.19±0.32 | 0.71±0.04 |
| Veratrole | 4.89±2.91 | 9.09±6.07 | 2.33±1.39 | 1.10±0.58 | 2.12±0.27 | 1.55±0.20 | 1.51±0.69 | 1.18±0.55 | 1.07±0.38 | 0.84±0.32 | 1.25±0.16 | 0.51±0.01 |
| Methoxyanisole | 2.75±1.75 | 2.64±1.42 | ND | ND | 0.31±0.07 | 0.87±0.19 | 0.49±0.17 | 0.32±0.11 | ND | 0.22±0.11 | ND | 0.24±0.03 |

Note: ND, means non detected.

**Table S4.** The FVOCs from the four organs of *N. profilera* mother-flowers.

| **Compounds (μg/g FW^-1^)** | **Sepal** | **Petal** | **Stamen** | **Pistil** |
| --- | --- | --- | --- | --- |
| Anisole | 2.76±1.85 | 115.35±86.58 | 889.71±523.48 | 438.01±274.49 |
| Veratrole | 4.84±1.08 | 37.61±20.01 | 105.76±102.04 | 11.80±6.29 |
| Methoxyanisole | 0.22±0.13 | 0.73±0.22 | 2.19±1.40 | 5.23±1.01 |
| Phenol | ND | 0.02±0.01 | ND | ND |
| Guaiacol | ND | 1.75±1.09 | 0.76±0.48 | 0.20±0.05 |
| Benzoic acid, methyl ester | ND | 0.17±0.03 | ND | ND |
| Benzyl alcohol | ND | ND | ND | 0.89±0.08 |
| Benzothiazole | ND | ND | ND | 0.58±0.52 |
| 4-Methoxybenzaldehyde | ND | ND | ND | 0.38±0.63 |
| α-Terpineol | ND | ND | 1.47±1.04 | ND |
| Caryophyllene | ND | ND | 0.89±1.50 | 2.44±1.15 |
| Pentadecane | 0.88±0.20 | 0.89±0.03 | ND | 0.77±0.26 |
| 6,9-Heptadecadiene | 0.06±0.04 | 0.11±0.10 | 0.27±0.19 | 0.89±1.39 |
| 8-Heptadecene | 0.13±0.12 | 0.19±0.16 | 0.27±0.14 | 0.31±0.37 |
| Heptadecane | ND | 0.03±0.01 | ND | 0.08±0.05 |
| Anisole ratio | 0.51±0.34 | 0.70±0.09 | 0.83±0.23 | 0.94±0.04 |
| Veratrole ratio | 0.27±0.17 | 0.28±0.09 | 0.17±0.12 | 0.05±0.02 |
| Methoxyanisole ratio | 0.04±0.02 | 0.0032±0.0012 | 0.0018±0.0024 | 0.0073±0.0125 |

Note: ND, means non detected.

**Table S5.** Summary of the FVOCs in waterlilies.

**Table S6.** The specific primers for gene expression analyzed by qRT-PCR for the four *NpOMTs*.

| Genes | Primers |
| --- | --- |
| *NproOMT1* | F: AGACTGCGTGAAGATACTG |
|  | R: TTCGTTGAACACTTGATGAG |
| *NproOMT2* | F: CATCACTACAACTACCTAACG |
|  | R: ACAACTTCTTCCATTCCTCT |
| *NproOMT3* | F: TACTGCCACCTCTCCTTC |
|  | R: TTGAATAACTTGCCGAATCC |
| *NproOMT4* | F: AAGCAATTCCACATGACGA |
|  | R: CTTCCTCCACTCTACTTCTG |
| *NproOMT5* | F: AGTGTGAAGCCAATGAAGA |
|  | R: CATTCTGCCTCTGTTCTCT |
| *NproOMT6* | F: GGCGTTGACCACAGTTAC |
|  | R: ATGACCCAAGAACCGTAGA |
| *NproOMT7* | F: CAGTCACGGCAGTAACAG |
|  | R: ACATCTTCTTGCGGAACTC |
| *NproOMT8* | F: AAGGTGATTGTTGTGGAGAT |
|  | R: ATCGGTGCTCTTGTTCTTAT |
| *NproOMT9* | F: AAGGTGGTCGTGGTTGAG |
|  | R: TGTAGACGCAGCAGATGG |
| *NproOMT10* | F: TGTTGAAGAACTGCTGGAA |
|  | R: CTGTTACTGCCGTGACTG |
| *NproOMT11* | F: CGTTGGTGGTGATATGTTC |
|  | R: GCGTAACTGTGGTCAATAAC |
| *NproOMT12* | F: CTTCAACGCATCCTCCAA |
|  | R: TCGCAGTATGTAATCACCAA |

**Table S7.** The detailed information of OMTs for polygenetic analysis.

| Abbreviation | Accession No. | Species | Substrates | References |
| --- | --- | --- | --- | --- |
| MdoOMT1 | AKN09016.1 | *Malus domestica* | Chavicol | 1 |
| EjGOMT | BAV54107.1 | *Eriobotrya japonica* | Guaiacol | 2 |
| NtCaOMT | CAA50561.1 | *Nicotiana tabacum* | Catechol | 3 |
| ObCVOMT1 | Q93WU3.1 | *Ocimum basilicum* | Chavicol | 4 |
| ObEOMT1 | Q93WU2.1 | *Ocimum basilicum* | Eugenol | 4 |
| TtCaOMT | AAD29844.1 | *Thalictrum tuberosum* | Catechol | 5 |
| SlCaOMT | NP_001306101.1 | *Solanum lycopersicum* | Catechol | 6 |
| RhOrOMT1 | AAM23004.1 | *Rosa hybrid cultivar* | Orcinol | 7 |
| RhOrOMT2 | AAM23005.1 | *Rosa hybrid cultivar* | Orcinol | 7 |
| RcEOMT | BAC78826.1 | *Rosa chinensis var. spontanea* | Eugenol | 8 |
| RcCaffOMT | BAC78827.1 | *Rosa chinensis var. spontanea* | Caffeic acid | 8 |
| RcCaffOMT1 | BAC78828.1 | *Rosa chinensis var. spontanea* | Caffeic acid | 8 |
| RcPhOMT | BAD18975.1 | *Rosa chinensis var. spontanea* | Phloroglucinol | 8 |
| MsCaffOMT | AAB46623.1 | *Medicago sativa* | Caffeic acid | 9 |
| MtIOMT7 | ABD83946.1 | *Medicago truncatula* | Isoflavone and Isoflavanone | 10 |
| RcOriOMT1 | Q8GU24 | *Rosa chinensis* | Orcinol | 11 |
| RcOriOMT2 | Q8GU23 | *Rosa chinensis* | Orcinol | 11 |
| VvResOMT | B6VJS4 | *Vitis vinifera* | Trans-resveratrol | 12 |
| CbIEOMT | AAC01533.1 | *Clarkia breweri* | Eugenol/Isoeugenol | 13 |
| NtCaffOMT | AAL91506.1 | *Nicotiana tabacum* | Caffeic acid | 14 |
| DcIs(E)OMT | XP_017238865.1 | *Daucus carota subsp. sativus* | Eugenol/Isoeugenol | 15 |
| GmInoOMT | NP_001242325.2 | *Glycine max* | Pinitol | 14 |
| GmIsoOMT | NP_001353843.1 | *Glycine max* | Isoflavone | 16 |
| SmCaffOMT | ADE88153.1 | *Selaginella moellendorffii* | Caffeyl alcohol/5-Hydroxyconiferyl alcohol | 17 |
| AmtCaOMT | XP_006829216.1 | *Amborella trichopoda* |  | 18,19 |
| AmtRetOMT1 | XP_011622314.1 | *Amborella trichopoda* |  | 18,19 |
| AmtRetOMT2 | XP_011622311.2 | *Amborella trichopoda* |  | 18,19 |
| AmtFlaOMT | XP_006838729.1 | *Amborella trichopoda* |  | 18,19 |
| AmtRetOMT3 | XP_006827606.2 | *Amborella trichopoda* |  | 18,19 |
| AmtNorOMT | XP_006858228.1 | *Amborella trichopoda* |  | 18,19 |
| AmtOMT2 | XP_006841695.3 | *Amborella trichopoda* |  | 18,19 |
| CbIEMT | AAB71141.1 | *Clarkia breweri* | (Iso)eugenol | 20 |
| AtCaffOMT | AAB96879.1 | *Arabidopsis thaliana* | Caffeic acid/5-hydroxyferulic acid | 21 |
| AtOMT1 | NP_200227.1 | *Arabidopsis thaliana* | Quercetin | 22 |
| SmCOMT | ADE88151.1 | *Selaginella moellendorffii* | Caffeyl alcohol/5-hydroxyconiferyl alcohol | 17 |
| GmSOMT-2 | C6TAY1.1 | *Glycine max* | Naringenin | 23 |
| GmIMT | NP_001243073.1 | *Glycine max* | 3-Hydroxy-N-methyl-(S)-coclaurine | 24 |
| VvROMT | NP_001268044.1 | *Vitis vinifera* | Resveratrol | 12 |
| PtAEOMT | AAC49708.1 | *Pinus taeda* | Caffeic and 5-hydroxyferulic acid | 25 |
| MsIOMT | AAC49928.1 | *Medicago sativa* | Isoflavones/(+) 6a-hydroxymaackiain | 26 |
| MsOMT | AAB48059.1 | *Medicago sativa* | Isoliquiritigenin | 27 |
| HiOMT | ABZ89566.1 | *Humulus lupulus* | Isoliquiritigenin/Resveratrol | 28 |
| HiDeOMT | ABZ89565.1 | *Humulus lupulus* | Desmethylxanthohumol | 28 |
| ZmFOMT2 | UED15621.1 | *Zea mays* | 2-Hydroxynaringenin | 29 |
| ZmFOMT4 | UED15622.1 | *Zea mays* | 3-Hydroxynaringenin | 29 |
| NcOMT1 | XP_031500854.1 | *Nymphaea colorata* |  | 30 |
| NcOMT13 | XP_031482634.1 | *Nymphaea colorata* |  | 30 |
| NcOMT114 | XP_031498623.1 | *Nymphaea colorata* |  | 30 |
| NcOMT15 | XP_031474277.1 | *Nymphaea colorata* |  | 30 |
| NcOMT16 | XP_031497294.1 | *Nymphaea colorata* |  | 30 |
| NcOMT17 | XP_031497292.1 | *Nymphaea colorata* |  | 30 |
| NcOMT18 | GWHTAAYW004586 | *Nymphaea colorata* |  | 30 |
| NcOMT19 | XP_031498584.1 | *Nymphaea colorata* |  | 30 |
| NcOMT20 | XP_031483824.1 | *Nymphaea colorata* |  | 30 |
| NcOMT21 | XP_031482634.1 | *Nymphaea colorata* |  | 30 |
| NcOMT22 | XP_031497294.1 | *Nymphaea colorata* |  | 30 |
| NcOMT23 | XP_031503202.1 | *Nymphaea colorata* |  | 30 |
| NcOMT24 | XP_031483824.1 | *Nymphaea colorata* |  | 30 |
| NcOMT2 | GWHTAAYW006192 | *Nymphaea colorata* |  | 30 |
| NcOMT3 | XP_031500700.1 | *Nymphaea colorata* |  | 30 |
| NcOMT25 | XP_031495262.1 | *Nymphaea colorata* |  | 30 |
| NcOMT26 | XP_031483825.1 | *Nymphaea colorata* |  | 30 |
| NcOMT27 | XP_031500345.1 | *Nymphaea colorata* |  | 30 |
| NcOMT28 | XP_031484095.1 | *Nymphaea colorata* |  | 30 |
| NcOMT29 | XP_031484096.1 | *Nymphaea colorata* |  | 30 |
| NcOMT30 | XP_031482260.1 | *Nymphaea colorata* |  | 30 |
| NcOMT31 | XP_031483180.1 | *Nymphaea colorata* |  | 30 |
| NcOMT32 | XP_031483179.1 | *Nymphaea colorata* |  | 30 |
| NcOMT33 | XP_031483595.1 | *Nymphaea colorata* |  | 30 |
| NcOMT4 | XP_031499301.1 | *Nymphaea colorata* |  | 30 |
| NcOMT5 | GWHPAAYW006185 | *Nymphaea colorata* |  | 30 |
| NcOMT6 | XP_031501213.1 | *Nymphaea colorata* |  | 30 |
| NcOMT7 | XP_031500345.1 | *Nymphaea colorata* |  | 30 |
| NcOMT8 | XP_031504677.1 | *Nymphaea colorata* |  | 30 |
| NcOMT9 | GWHTAAYW009296 | *Nymphaea colorata* |  | 30 |
| NcOMT10 | XP_031503788.1 | *Nymphaea colorata* |  | 30 |
| NcOMT11 | XP_031482942.1 | *Nymphaea colorata* |  | 30 |
| NcOMT12 | XP_031473063.1 | *Nymphaea colorata* |  | 30 |
|  | A0A2K3DVI9 | *Chlamydomonas reinhardtii* |  | 31 |
|  | A0A8J4C985 | *Volvox reticuliferus* |  | 32 |
|  | A0A150GAC3 | *Gonium pectorale* |  | 33 |
|  | A0A0D2LDN7 | *Monoraphidium neglectum* |  | 34 |
|  | A0A8J9WQS0 | *Coccomyxa sp. Obi* |  | 35 |
|  | A0A8J9S8V1 | *Coccomyxa sp. Obi* |  | 35 |

**References**

1. Yauk, Y.K. *et al.* The *O*-methyltransferase gene *MdoOMT1* is required for biosynthesis of methylated phenylpropenes in ripe apple fruit. *The Plant Journal* **82**, 937-950 <http://dx.doi.org/10.1111/tpj.12861> (2015).

2. Koeduka, T. *et al.* Characterization of an *O*-methyltransferase specific to guaiacol-type benzenoids from the flowers of loquat (*Eriobotrya japonica*). *Journal of Bioscience and Bioengineering* **122**, 679-684 <http://dx.doi.org/10.1016/j.jbiosc.2016.06.012> (2016).

3. Pellegrini, L., Ceoffroy, P., Fritig, B. & Legrand, M. Molecular cloning and expression of a new class of ortho-diphenol-*O*-methyltransferases induced in tobacco (*Nicotiana tabacum* L.) leaves by infection or elicitor treatment. *Plant PhysioIogy* **103**, 509-517 (1993).

4. Gang, D.R. *et al.* Characterization of phenylpropene *O*-methyltransferases from sweet basil: facile change of substrate specificity and convergent evolution within a plant *O*-methyltransferase family. *Plant Cell* **14**, 505-519 <http://dx.doi.org/10.1105/tpc.010327> (2002).

5. Frick, S. & Kutchan, T.M. Molecular cloning and functional expression of *O*-methyltransferases common to isoquinoline alkaloid and phenylpropanoid biosynthesis. *The Plant Journal* **17**, 329–339. (1999).

6. Mageroy, M.H., Tieman, D.M., Floystad, A., Taylor, M.G. & Klee, H.J. A *Solanum lycopersicum* catechol-*O*-methyltransferase involved in synthesis of the flavor molecule guaiacol. *The Plant Journal* **69**, 1043-1051 <http://dx.doi.org/10.1111/j.1365-313X.2011.04854.x> (2012).

7. Lavid, N. *et al.* *O*-methyltransferases involved in the biosynthesis of volatile phenolic derivatives in rose petals. *Plant PhysioIogy* **129**, 1899-1907 <http://dx.doi.org/10.1104/pp.005330> (2002).

8. Wu, S. *et al.* Two *O*-methyltransferases isolated from flower petals of *Rosa chinensis* var. *spontanea* involved in scent biosynthesis. *Journal of Bioscience and Bioengineering* **96**, 119-128. (2003).

9. Gowri, G., Bugos, R.C., Campbell, W.H., Maxwell, C.A. & Dixon, R.A. Stress responsesin Alfalfa (*Medicago sativa* L.) X. molecular cloning and expression of S-adenosyl-L-methionine caffeic acid 3-*O*-methyltransferase, a key enzyme of lignin biosynthesis. *Plant PhysioIogy* **97**, 7-14 (1991).

10. Deavours, B.E. *et al.* Functional analysis of members of the isoflavone and isoflavanone *O*-methyltransferase enzyme families from the model legume *Medicago truncatula*. *Plant Molecular Biology* **62**, 715-733 <http://dx.doi.org/10.1007/s11103-006-9050-x> (2006).

11. Scallieta, G. *et al.* Biosynthesis of the major scent components 3,5-dimethoxytoluene and 1,3,5-trimethoxybenzene by novel rose *O*-methyltransferases. *FEBS Letters* **62**, 113-118 (2006).

12. Schmidlin, L. *et al.* A stress-inducible resveratrol *O*-methyltransferase involved in the biosynthesis of pterostilbene in grapevine. *Plant PhysioIogy* **148**, 1630-1639 <http://dx.doi.org/10.1104/pp.108.126003> (2008).

13. Wang, J., Dudareva, N., Bhakta, S., Raguso, R.A. & Pichersky, E. Floral scent production in *Clarkia breweri* (Onagraceae) II. localization and developmental modulation of the enzyme S-adenosyl-L-Methionine:(lso)Eugenol *O*-Methyltransferase and phenylpropanoid emission. *Plant PhysioIogy* **114**, 213-221 (1997).

14. Toquin, V., Grausem, B., Geoffroy, P. & Legrand, M. Structure of the tobacco caffeic acid *O*-methyltransferase (COMT) II gene: identification of promoter sequences involved in gene inducibility by various stimuli. *Plant Molecular Biology* **52**, 495–509 (2003).

15. Yahyaa, M. *et al.* Biosynthesis of methyleugenol and methylisoeugenol in *Daucus carota* leaves: characterization of eugenol/isoeugenol synthase and *O*-methyltransferase. *Phytochemistry* **159**, 179-189 <http://dx.doi.org/10.1016/j.phytochem.2018.12.020> (2019).

16. Uchida, K. *et al.* Identification of a unique type of isoflavone *O*-methyltransferase, GmIOMT1, based on multi-omics analysis of soybean under biotic stress. *Plant Cell Physiology* **61**, 1974-1985 <http://dx.doi.org/10.1093/pcp/pcaa112> (2020).

17. Weng, J.K., Akiyama, T., Ralph, J. & Chapple, C. Independent recruitment of an *O*-methyltransferase for syringyl lignin biosynthesis in *Selaginella moellendorffii*. *Plant Cell* **23**, 2708-2724 <http://dx.doi.org/10.1105/tpc.110.081547> (2011).

18. Amborella Genome, P. The Amborella genome and the evolution of flowering plants. *Science* **342**, 1241089 <http://dx.doi.org/10.1126/science.1241089> (2013).

19. Wu, S., Wilson, A.E., Chang, L. & Tian, L. Exploring the phytochemical landscape of the early-diverging flowering plant *Amborella trichopoda* Baill. *Molecules* **24**, 3814 <http://dx.doi.org/10.3390/molecules24213814> (2019).

20. Wang, J. & Pichersky, E. Characterization of S-adenosyl-L-methionine:(Iso)eugenol *O*-methyltransferase involved in floral scent production in *Clarkia breweri*. *Archives of Biochemistry and Biophysics* **349**, 153–160. (1998).

21. Zhang, H., Wang, J. & Goodman, H.M. An *Arabidopsis* gene encoding a putative 14-3-3-interacting protein, caffeic acid/5-hydroxyferulic acid *O*-methyltransferase. *Biochimica et Biophysica Acta* **1353**, 199–202 (1997).

22. Fellenberg, C., Ohlen, M.V., Handrick, V. & Vogt, T. The role of CCoAOMT1 and COMT1 in *Arabidopsis* anthers. *Planta* **236**, 51-61 <http://dx.doi.org/10.1007/s00425-011-1586-6> (2012).

23. Kim, D.H. *et al.* Regiospecific methylation of naringenin to ponciretin by soybean *O*-methyltransferase expressed in *Escherichia coli*. *Journal of Biotechnology* **119**, 155-162 <http://dx.doi.org/10.1016/j.jbiotec.2005.04.004> (2005).

24. Ahn, C., Park, U. & Park, P.B. Increased salt and drought tolerance by D-ononitol production in transgenic *Arabidopsis thaliana*. *Biochemical and Biophysical Research Communications* **415**, 669-674 <http://dx.doi.org/10.1016/j.bbrc.2011.10.134> (2011).

25. Li, L. *et al.* A novel multifunctional *O*-methyltransferase implicated in a dual methylation pathway associated with lignin biosynthesis in loblolly pine. *Proceedings of the National Academy of Sciences, USA* **94**, 5461–5466 (1997).

26. He, X.Z., Reddy, J.T. & Dixon, R.A. Stress responses in alfalfa (*Medicago sativa* L). XXII. cDNA cloning and characterization of an elicitor-inducible isoflavone 7-*O*-methyltransferase. *Plant Molecular Biology* 43–54 (1998).

27. Maxwell, C.A., Harrison, M.J. & Dixon, R.A. Molecular characterization and expression of alfalfa isoliquiritigenin 2'-*O*-methyltransferase, an enzyme specifically involved in the biosynthesis of an inducer of *Rhizobium meliloti* nodulation genes. *The Plant Journal* **4**, 971-981 (1993).

28. Nagel, J. *et al.* EST analysis of hop glandular trichomes identifies an *O*-methyltransferase that catalyzes the biosynthesis of xanthohumol. *Plant Cell* **20**, 186-200 <http://dx.doi.org/10.1105/tpc.107.055178> (2008).

29. Förster, C. *et al.* Biosynthesis and antifungal activity of fungus-induced *O*-methylated flavonoids in maize. *Plant PhysioIogy* **188**, 167-190 <http://dx.doi.org/10.1093/plphys/kiab496> (2022).

30. Zhang, L. *et al.* The water lily genome and the early evolution of flowering plants. *Nature* **577**, 79-84 <http://dx.doi.org/10.1038/s41586-019-1852-5> (2019).

31. Merchant, S.S. *et al.* The *Chlamydomonas* genome reveals the evolution of key animaland plant functions. *Science* **318**, 245-250 (2007).

32. Yamamoto, K. *et al.* Three genomes in the algal genus Volvox reveal the fate of a haploid sex-determining region after a transition to homothallism. *Proc Natl Acad Sci U S A* **118**, <http://dx.doi.org/10.1073/pnas.2100712118> (2021).

33. Hanschen, E.R. *et al.* The *Gonium pectorale* genome demonstrates co-option of cell cycle regulation during the evolution of multicellularity. *Nat Commun* **7**, 11370 <http://dx.doi.org/10.1038/ncomms11370> (2016).

34. Bogen, C. *et al.* Reconstruction of the lipid metabolism for the microalga Monoraphidium neglectum from its genome sequence reveals characteristics suitable for biofuel production. *BMC Genomics* **14**, (2013).

35. Harayama, S. The genome sequence of the unicellular green alga, *Coccomyxa sp. strain Obi.* (2021).
